# Electrically controlled transformation of memristive titanates into mesoporous titanium oxides via incongruent sublimation

**DOI:** 10.1038/s41598-018-22238-4

**Published:** 2018-02-28

**Authors:** C. Rodenbücher, P. Meuffels, G. Bihlmayer, W. Speier, H. Du, A. Schwedt, U. Breuer, C.-L. Jia, J. Mayer, R. Waser, K. Szot

**Affiliations:** 1 Peter Grünberg Institute, Forschungszentrum Jülich GmbH, 52425 Jülich, Germany; 2 JARA - Fundamentals of Future Information Technologies, Forschungszentrum Jülich GmbH, 52425 Jülich, Germany; 3 Institute of Advanced Simulation, Forschungszentrum Jülich, 52425 Jülich, Germany; 4 Ernst Ruska-Centre for Microscopy and Spectroscopy with Electrons, Forschungszentrum Jülich GmbH, 52425 Jülich, Germany; 5 Gemeinschaftslabor für Elektronenmikroskopie, RWTH Aachen, 52056 Aachen, Germany; 6 Central Institute for Engineering, Electronics and Analytics, Forschungszentrum Jülich GmbH, 52425 Jülich, Germany; 7 Institut für Werkstoffe der Elektrotechnik 2, RWTH Aachen, 52056 Aachen, Germany; 8 A. Chełkowski Institute of Physics, University of Silesia, 40-007 Katowice, Poland

## Abstract

Perovskites such as SrTiO_3_, BaTiO_3_, and CaTiO_3_ have become key materials for future energy-efficient memristive data storage and logic applications due to their ability to switch their resistance reversibly upon application of an external voltage. This resistance switching effect is based on the evolution of nanoscale conducting filaments with different stoichiometry and structure than the original oxide. In order to design and optimize memristive devices, a fundamental understanding of the interaction between electrochemical stress, stoichiometry changes and phase transformations is needed. Here, we follow the approach of investigating these effects in a macroscopic model system. We show that by applying a DC voltage under reducing conditions on a perovskite slab it is possible to induce stoichiometry polarization allowing for a controlled decomposition related to incongruent sublimation of the alkaline earth metal starting in the surface region. This way, self-formed mesoporous layers can be generated which are fully depleted by Sr (or Ba, Ca) but consist of titanium oxides including TiO and Ti_3_O with tens of micrometre thickness. This illustrates that phase transformations can be induced easily by electrochemical driving forces.

## Introduction

As promising materials for novel electronic but also catalytic applications, ternary titanates with perovskite structure have been widely investigated. In particular, the discovery of the resistive switching effect has generated enormous interest as it can be exploited for future memristive data storage and neuromorphic computing^1^. For the investigation of the performance of ternary metal oxides, strontium titanate (SrTiO_3_) has become a prototype material due to the possibility of controlling its resistance between insulating, metallic and even superconducting behaviour related to a self-doping effect by oxygen vacancies^2–5^. While oxides with perovskite structure are regarded as very stable materials since many of them can be found in rocks, it has been shown recently that resistive switching is related to a local decomposition and phase transformation by an electroforming process resulting in the evolution of switchable nanofilamentary structures^6,7^. As the main driving force of this transformation, the movement of oxygen vacancies upon application of an electric field has been identified. Inspired by this nanoscale mechanism, here, we investigate the behaviour of perovskites under high electrochemical stress by following the well-established approach of macroscopic electrodegradation^8–10^ to make phase transformations upon current flow visible, focusing on the surface region. In general, two channels of surface decomposition of a solid compound exist, namely segregation and incongruent sublimation^11^. While segregation effects are known to occur upon thermal annealing only to limited extent^12,13^, we have shown recently^14^ that macroscopic decomposition of titanates via incongruent sublimation of the alkaline earth metal can occur at extremely low oxygen activity even at relatively moderate temperatures (>650 °C). In the present study, we now give evidence that phase decomposition in the surface region related to incongruent sublimation can be controlled upon the application of an electric field causing a movement of oxygen from the cathode region towards the anode region. This results in a difference of ionic concentration which is called “stoichiometry polarization”^15,16^. We demonstrate that the surface region of the ternary oxides SrTiO_3_, BaTiO_3_, and CaTiO_3_ can be transformed into lower binary oxides by means of electrodegradation under vacuum conditions (*p* < 10^−6^ mbar). Based on detailed measurements of the chemical composition, the electronic structure and the crystallographic structure, we propose that by the in-plane stoichiometry polarization the oxygen activity at the cathode region is significantly reduced such that the conditions for incongruent sublimation are fulfilled and an out-of-plane decomposition takes place. Hence, the alkaline earth metal (Sr, Ba, or Ca) sublimates from the cathode region and substoichiometric titanium oxide phases evolve at the surface changing the physical properties of the surface region up to depths of several tens of micrometres. Our results indicate that in ternary oxides phase transformations can be generated by gradients of the electrochemical potential using a technologically simple method, which not only opens up a new path for tailoring microlayers of functional transition metal oxides with bespoke properties for optical, electronic and chemical applications, but also demonstrates the mutability of metal oxides under electrical stress, which is relevant for understanding the electroforming and switching process in memristive devices.

## Results

**Figure 1:**
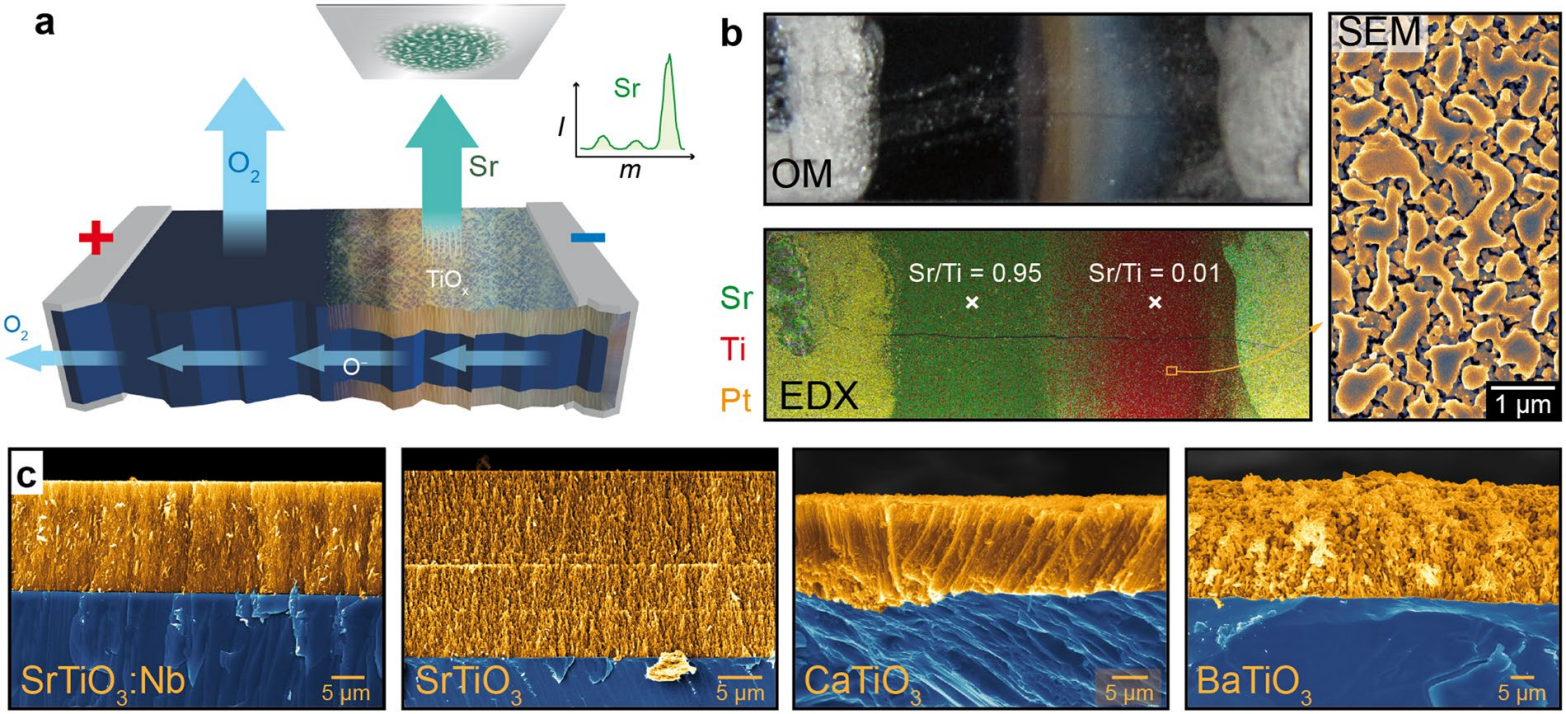
(**a**) Outline of the DC polarization. The inset shows Sr sublimation revealed by mass spectrometry. (**b**) Optical microscopy and EDX mapping of the decomposed DC sample with SEM images of the surface in the cathode region after decomposition. (**c**) Colorized cross-sections of the mesoporous layer on different perovskite-type oxides.

The setup of the conducted electrodegradation experiment is shown schematically in Fig. 1a. As reference sample, we investigated the commonly used and commercially available conducting oxide SrTiO_3_:Nb (0.7 wt%) in form of a single crystal that we contacted using pasted Pt-electrodes at the end faces. Due to the porous structure of the Pt, the electrodes themselves were transparent for oxygen exchange with the ambient. A DC voltage of max. 14 V was applied under vacuum conditions (*p* < 10^−6^ mbar) with a current compliance of 2.2 A which heated the sample via Joule heating leading to thermal reduction and additionally established a stoichiometry polarization by causing an oxygen movement from cathode towards anode (for details of this mechanism see discussion below). This way, a low oxygen activity in the surface layer of the cathode region was established and the sublimation of Sr was observed as can be seen by mass spectrometry (Fig. 1a, green line). After the treatment, a macroscopic decomposition of the cathode region was evident even by simple optical inspection revealing that a colour change from originally black towards reddish to greyish stripes had occurred (Fig. 1b, top). EDX mapping of the surface confirmed that the cathode region (right) has been depleted by Sr while the anode region (left) has not changed significantly and still showed a chemical composition typically of SrTiO_3_ (Fig. 1b, bottom). Also in close vicinity to the cathode, no significant decomposition was observed. This can be explained by the relatively low temperature (<500 °C) established since the electrical contacts served simultaneously as heat sinks. To verify that this indeed is a temperature effect, we heated the whole sample externally via a tube furnace in a control electrodegradation experiment and found also a decomposition below the Pt electrode of the cathode as illustrated in Fig. 1a. The decomposed surface in the cathode region consisted of crystallites with diameters ranging from tens of nanometres up to a few micrometres forming a porous structure (Fig. 1b, right). A cross section of the cathode region was performed by mechanical cleaving and subsequent inspection of the cleavage plane by SEM/EDX. As shown in Fig. 1c, the nanoporous layer that was found to be almost completely depleted by Sr had a thickness up to several tens of micrometres while the bulk of the material had not changed significantly still showing a Sr/Ti ratio close to stoichiometric SrTiO_3_:Nb. Between the porous layer and the bulk of the crystal a very sharp interface can be seen. To illustrate that the electrochemical generation of nanoporous TiO_x_ surface layers is a general effect that can be induced on various ternary oxides we repeated the experiment using undoped SrTiO_3_ (single crystal), the original perovskite CaTiO_3_ (ceramic) and ferroelectric BaTiO_3_ (single crystal). These materials were annealed under reducing vacuum conditions (*p* < 10^−6^ mbar) at 1000 °C to induce a sufficient high electronic conductivity via self-doping by oxygen vacancies prior to the electrodegradation. In all cases, we found the same trend with sublimation of the alkaline earth metal during DC polarization and the creation of a nanoporous TiO_x_ layer in the cathode region (Fig. 1c).

**Figure 2:**
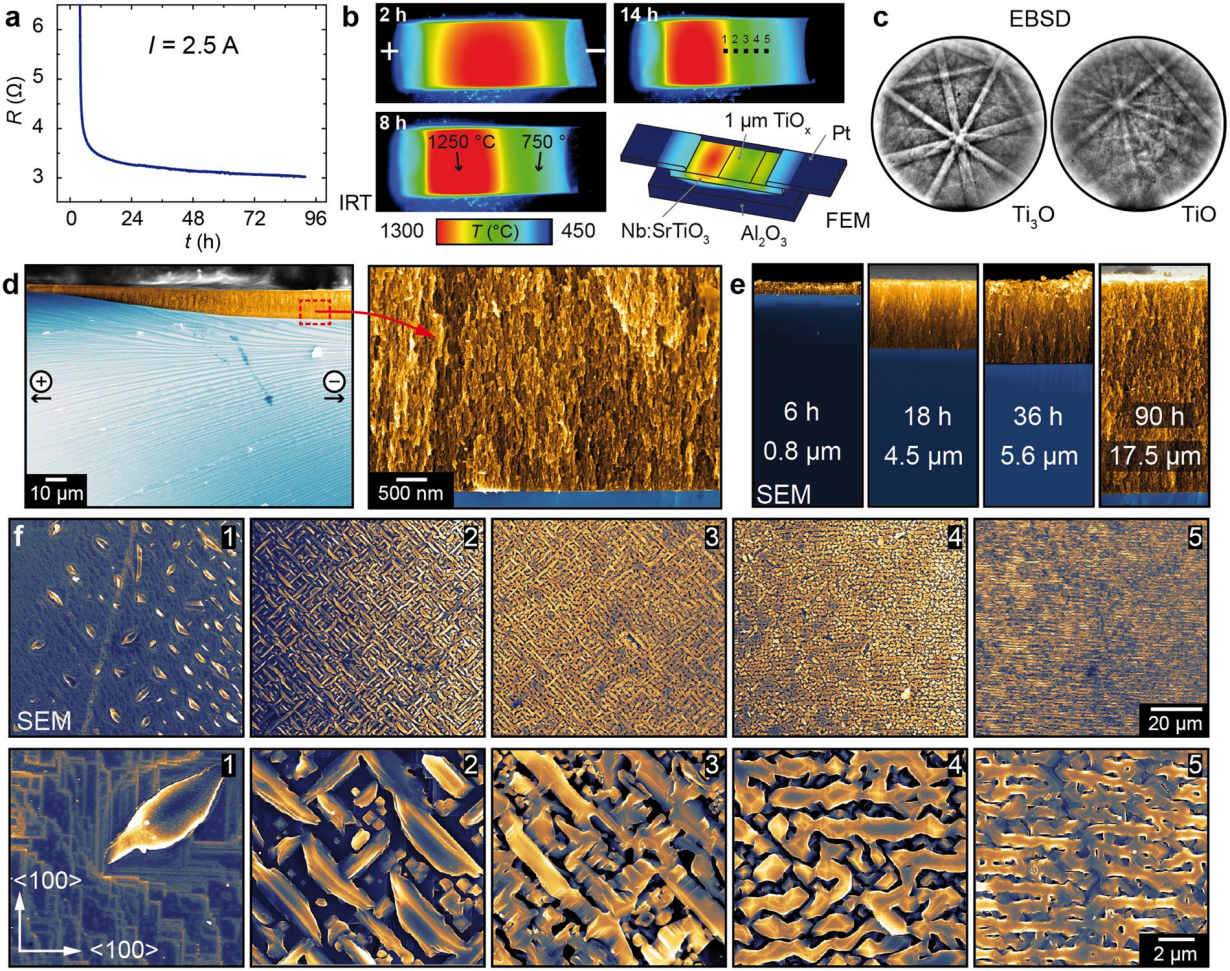
Analysis of the decomposed surface. (**a**) Overall resistance as function of degradation time. (**b**) Temperature distribution obtained by thermography during electrodegradation and FEM temperature simulation. (**c**) EBSD analysis of the surface in the cathode region. (**d**) SEM cross section of the cathode region. (**e**) SEM cross sections for different degradation times. (**f**) SEM top view at different positions at the cathode side marked in (**b**) The bottom row displays magnifications of the upper images.

In the following, we focus on the details of the decomposition during electrodegradation using the example of SrTiO_3_:Nb. At first we present the degradation of the total resistance of the sample during electrodegradation. We conducted this part of the experiment at a current compliance of 2.5 A resulting in a decrease of voltage with decreasing resistance. In the beginning of the degradation process, the 7 × 3 × 0.5 mm^3^ sample had a resistance of several tens of Ohm, mainly related to the existence of an insulating interface layer on pristine SrTiO_3_:Nb^3^, which dropped to approx. 3 Ω after 90 h of degradation (Fig. 2a). During this process, the Joule heating effect upon DC current flow was monitored by an infrared camera (cf. Fig. 2b and Supplementary Video). In the first stage of electrodegradation, the temperature distribution was symmetrical with a maximum temperature around 1300 °C located at the centre of the sample between the two electrodes. The temperature at the electrodes themselves was significantly lower (<500 °C) since the electrical connections attached to the electrodes served as a heat sink. As soon as the decomposition started, it can be seen that the maximum temperature shifted to the anode side (left-hand side in Fig. 2b) while the cathode side ended up well below 900 °C. At longer degradation times, the asymmetry in temperature distribution became pronounced and the evolution of the striped pattern of the transformed region also became visible in the infrared image. The dimension of the evolving TiO_x_ layer depends strongly on the applied power which determines the maximum temperature and the temperature distribution. For example, when the current compliance was adjusted to 3 A, the region of maximum temperature was shifted completely to the anode and almost the entire surface region transformed into mesoporous TiO_x_ with a thickness of more than 20 µm.

In order to understand the shift of the temperature distribution upon electrodegradation, we simulated the temperature in the stage of decomposition when the decomposed layer in the cathode region already has evolved using the finite element method (FEM) implemented in the Ansys software. We modelled a SrTiO_3_:Nb crystal contacted with Pt electrodes and supported by an Al_2_O_3_ holder. The resistivity of the crystal (*ρ*_*c*_ = 3∙10^−4^ Ωm) was chosen according to the measured values (*U* = 8 V, *I* = 2 A). On the surface of the crystal in the cathode region, we modelled a 1 µm thick metallic layer (*ρ*_*l*_ = 5∙10^−6^ Ωm) representing the decomposed TiO_x_ region. In the simulation, the same thermal conductivity (*λ* = 12 W/(m∙K)) for crystal and decomposed layer was used. This way, the layer short-circuits the cathode region which results in a shift of the majority of the potential drop towards the anode region. The calculation of the resulting temperature shown in Fig. 2b reproduces the measured asymmetric temperature distribution with the maximum located at the anode side giving a reasonable explanation for the observed temperature shift. The crystallographic structure of the decomposed surface was investigated by EBSD. On the surface different crystallites were found and we were able to identify the Kikuchi pattern of TiO and Ti_3_O, which is known to be particularly stable in Nb-doped systems^17^, as shown in Fig. 2c and Supplementary Information Ie. Using SEM inspection after mechanical cleaving, we investigated the thickness of the TiO_x_ layer. As shown in the overview image (Fig. 2d) obtained in the centre of the sample (corresponding to position 1 in Fig. 2b) it can be seen that the thickness of the layer increases from the left (anode side) towards the right (cathode side). Remarkably, the interface between the TiO_x_ layer and the bulk still maintaining the SrTiO_3_:Nb structure is extremely sharp even on the nanoscale as shown in the magnification of Fig. 2d. The maximum thickness of the nanoporous TiO_x_ layer evolving in the cathode region increases with the electrodegradation time as can be seen from the cross sections of crystals degraded for different times under the same conditions (Fig. 2e and Supplementary Information Ia). The displayed value is the maximum thickness of each layer determined with an error of approx. 0.1 µm. While in the first hours of degradation no significant changes can be observed, after 6 h, the layer evolution gets visible reaching tens of micrometre maximum thickness after several days. This shows that the decomposition can be regarded as a continuous process taking place at the interface between layer and bulk crystal. In contrast to the slow decomposition of Nb-doped SrTiO_3_, the evolution of the nanoporous layer on pre-reduced undoped SrTiO_3_ is significantly faster and already after one hour, a TiO_x_ layer with micrometre thickness evolves at comparable current density (for details see Supplementary Information III). This can be understood taking into account that the ionic conductivity of donor-doped SrTiO_3_ is significantly lower than for undoped SrTiO_3_ and thus more time is needed to establish a sufficient stoichiometry polarisation. The crystallites exhibit a characteristic structure and orientation as can be seen in the SEM top views (Fig. 2f) obtained from different positions in the cathode region marked in Fig. 2b. In the centre of the sample (position 1) only single TiO_x_ crystallites can be seen embedded in the SrTiO_3_ surrounding. Closer to the cathode, the density of crystallites increases and finally the whole surface is covered with TiO_x_. The crystallites are not randomly distributed but they are closely related to the crystallographic structure of the substrate. In particular in the low-density area, it can be seen that they are oriented along the 45° direction with respect to the <100> orientation of the crystal. This indicates that the distribution of extended defects such as dislocations which also tend to agglomerate along the crystallographic directions influences the evolution of the crystallites e.g. by serving as seeds (for details see Supplementary Information III).

**Figure 3:**
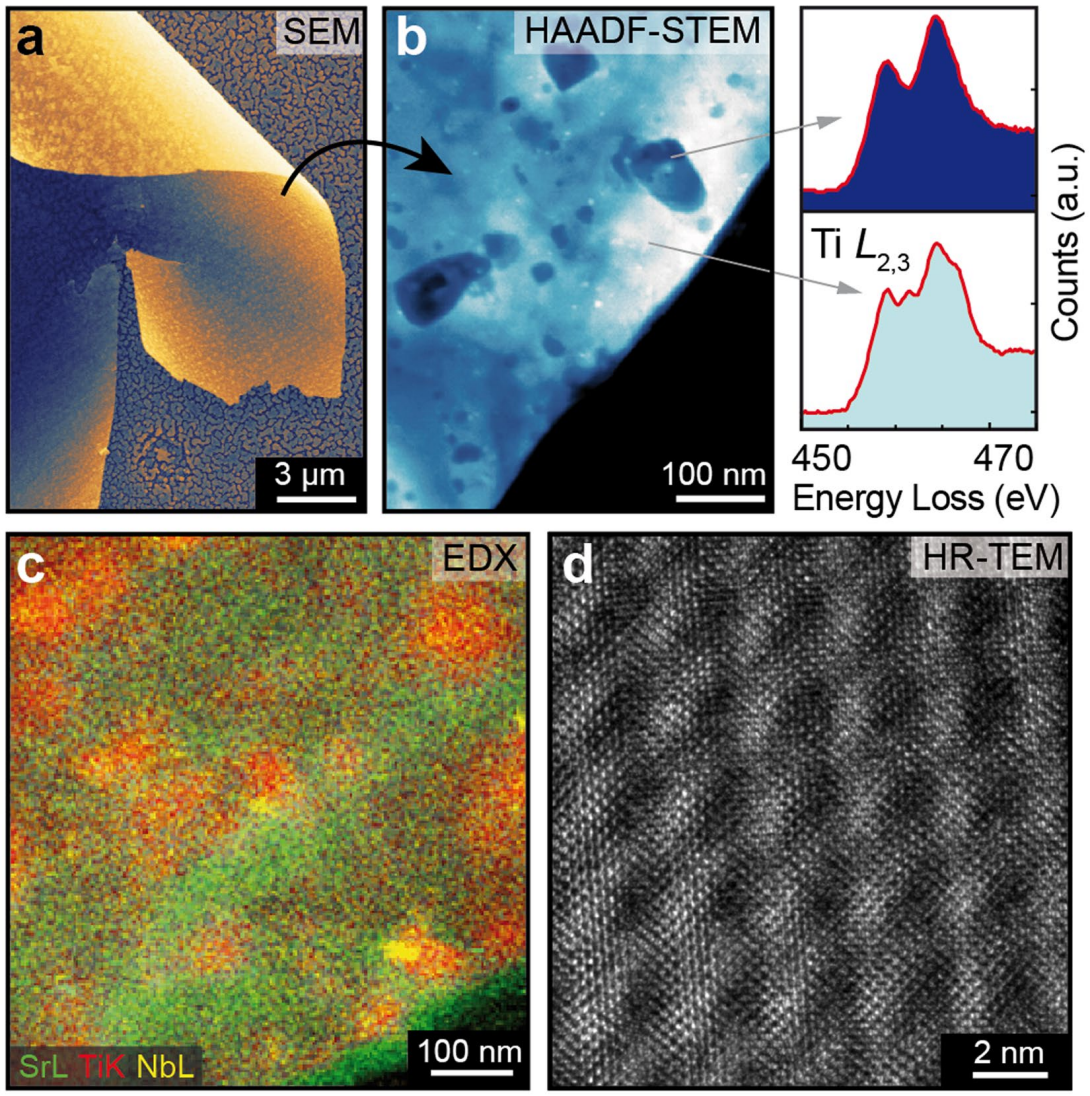
The evolved TiO_x_ layer. (**a**) SEM images of partly delamination of the layer, (**b**) HAADF-STEM images and EELS spectra of a flake of the delaminated layer, (**c**) EDX element mapping, (**d**) HR-TEM image.

Occasionally, we observed that the top of the TiO_x_ layer was able to delaminate itself completely or in part from the sample, probably due to the different thermal expansion during heating and subsequent cooling. This gave us the opportunity to investigate the properties of the layer by transmission electron microscopy without further preparation (Fig. 3a). Flakes of the delaminated self-supporting layer with a thickness of approx. 60 nm were analysed by high-angle annular dark-field scanning transmission electron microscopy (HAADF-STEM, Fig. 3b) and an inhomogeneous structure of TiO_x_ nanoclusters with diameters of 20–100 nm was observed. Inside the clusters, the spectrum of the TiL_2,3_ edge measured by electron energy loss spectroscopy (EELS) revealed the valence state +2, whereas a secondary valence was present outside the clusters^18^ illustrating phase separation on the nanoscale. Additionally, a segregation of all elements took place and especially clusters with Ti enrichment and in some positions small Nb clusters were found by EDX (Fig. 3c). The crystallographic structure of the delaminated flake was analysed by HR-TEM (Fig. 3d). A moiré pattern was recorded that could be described by an interference of the perovskite structure and the cubic TiO structure indicating that upon the fundamental phase transformation induced by electrodegradation a coexistence of phases is achieved.

**Figure 4:**
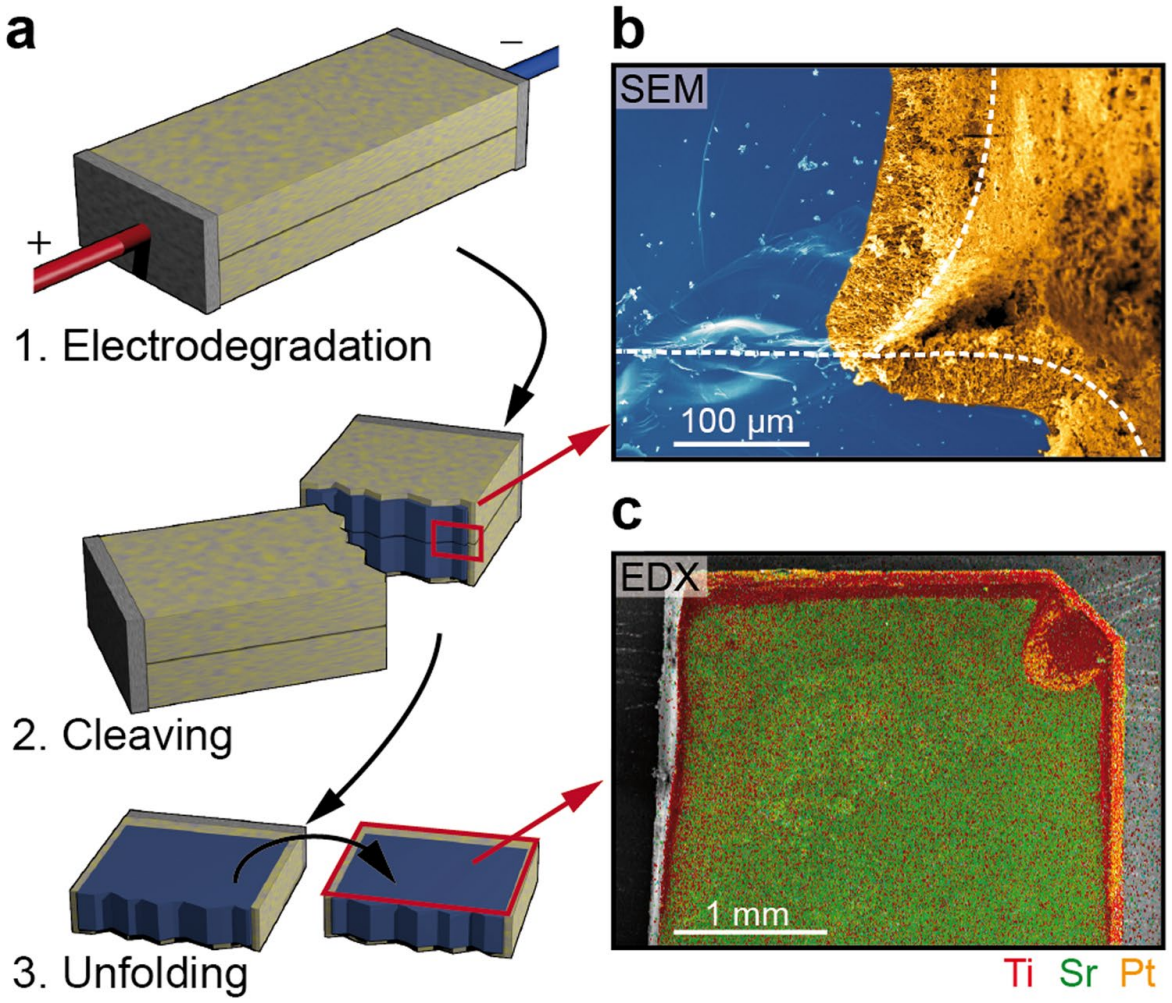
Electrodegradation of a sandwich structure of undoped SrTiO_3_. (**a**) Illustration of the procedure. (**b**) SEM analysis of the cleaved sandwich (the white dashed lines mark the edge of the samples, the yellowish part on the right of the line is just the outer face of the sample seen under the perspective of the SEM). (**c**) EDX map of the unfolded inner surface.

To elucidate the mechanism of decomposition and the influence of the surface, a control electrodegradation experiment in sandwich geometry was performed. Two crystals of SrTiO_3_ were stacked and contacted with pasted porous Pt contacts as non-blocking electrodes on their front sides as illustrated in Fig. 4. Here, we used undoped SrTiO_3_ for illustration since the decomposition was more efficient than in Nb-doped SrTiO_3_. The electrodegradation was performed by applying a voltage (*U*_*max*_ = 14 V, *I*_*cc*_ = 2.2 A) under vacuum conditions (*p* < 10^−6^ mbar) while the sample was externally heated to 1000 °C in a tube furnace. After the electrodegradation was performed for 12 h, the sample was cleaved and the cleavage plane was analysed by SEM. It can be seen (Fig. 4) that a nanoporous layer with morphology corresponding to the experiments described above only evolved on the outer surfaces of the crystals facing towards the vacuum while the inner surfaces did not change. Since the edges of the crystals were not ideally aligned during the electrodegradation, we marked the edges of the two samples by white dashed lines as guideline for the eye in Fig. 4b. To confirm that the inner surfaces had not decomposed, the stack was unfolded and analysed by SEM and EDX. In the centre of the inner surface, a Sr/Ti ratio close to 1 was measured typically of SrTiO_3_ and the morphology was still typically of an epi-polished crystal showing that no decomposition had taken place there. Only on the rims of the crystal that were facing to the vacuum, a Ti-rich layer was detected. Using this result, we can state that a free surface is needed for decomposition which indicate that the physical mechanism is indeed related to sublimation effects.

## Discussion

Summarizing our experiments, we found that when an alkaline earth titanate such as SrTiO_3_ is exposed to in-plane gradients of the electrical and electrochemical potential at moderately high temperatures (<1000 °C in the cathode region), an out-of-plane decomposition of the surface takes place in the cathode region. We confirmed that the decomposition results from the preferential sublimation of the alkaline earth metal leaving behind a porous TiO_x_-rich surface layer. In the following, we will present our qualitative understanding of the main mechanism leading to the surface decomposition although we are aware that the system is very complex because of a mutual interplay/coupling of various in-plane and out-of-plane thermal and thermodynamic potential gradients and transport processes.

At first, we would like to briefly discuss mechanisms of decomposition under electrochemical stress that are presented in literature and explain why we can exclude these effects in our setup. In principle, three mechanisms have to be considered namely kinetic demixing/decomposition^19^, solid state electrolysis^20^, and thermophoresis (Ludwig-Soret effect)^21^. In the case of kinetic demixing, a gradient of an electrochemical potential could lead to an in-plane decomposition due to of different cation mobilities. This was, e.g., found for NiTiO_3_ separating into NiO and TiO when exposed to a potential gradient^22^. For alkaline earth metal based titanates such as BaTiO_3_ instead, it has been concluded that kinetic decomposition does not occur because Ba^2+^ and Ti^4+^ have comparable electrochemical mobilities^23^. This stands in agreement with our observation that no in-plane decomposition, e.g. an agglomeration of SrO and TiO at anode and cathode, respectively, was observed, neither for doped nor for undoped SrTiO_3_. The second mechanism that we can exclude is solid state electrolysis. Regarding the experiment in “sandwich” geometry where we polarized two crystals in parallel directly touching each other (cf. Fig. 4) we observed that a decomposition only took place at the free surface while the inner surface did not show any significant decomposition. For decomposition by means of solid state electrolysis, it would not be relevant if a surface is facing vacuum or not; we thus conclude that solid state electrolysis is not the cause for the observed decomposition, but rather some sublimation effects. At third we focus on the Ludwig-Soret effect. As we have seen in Fig. 2b, the direct DC current flow causes a non-uniform Joule heating with a temperature gradient of several hundred degrees between anode and cathode. To exclude that thermophoresis plays an important role for the decomposition, we conducted an experiment where we generated a comparable temperature gradient using AC heating of a sample with asymmetric geometry and did not observe any decomposition or Sr evaporation (cf. Supplementary Information Ic).

**Figure 5:**
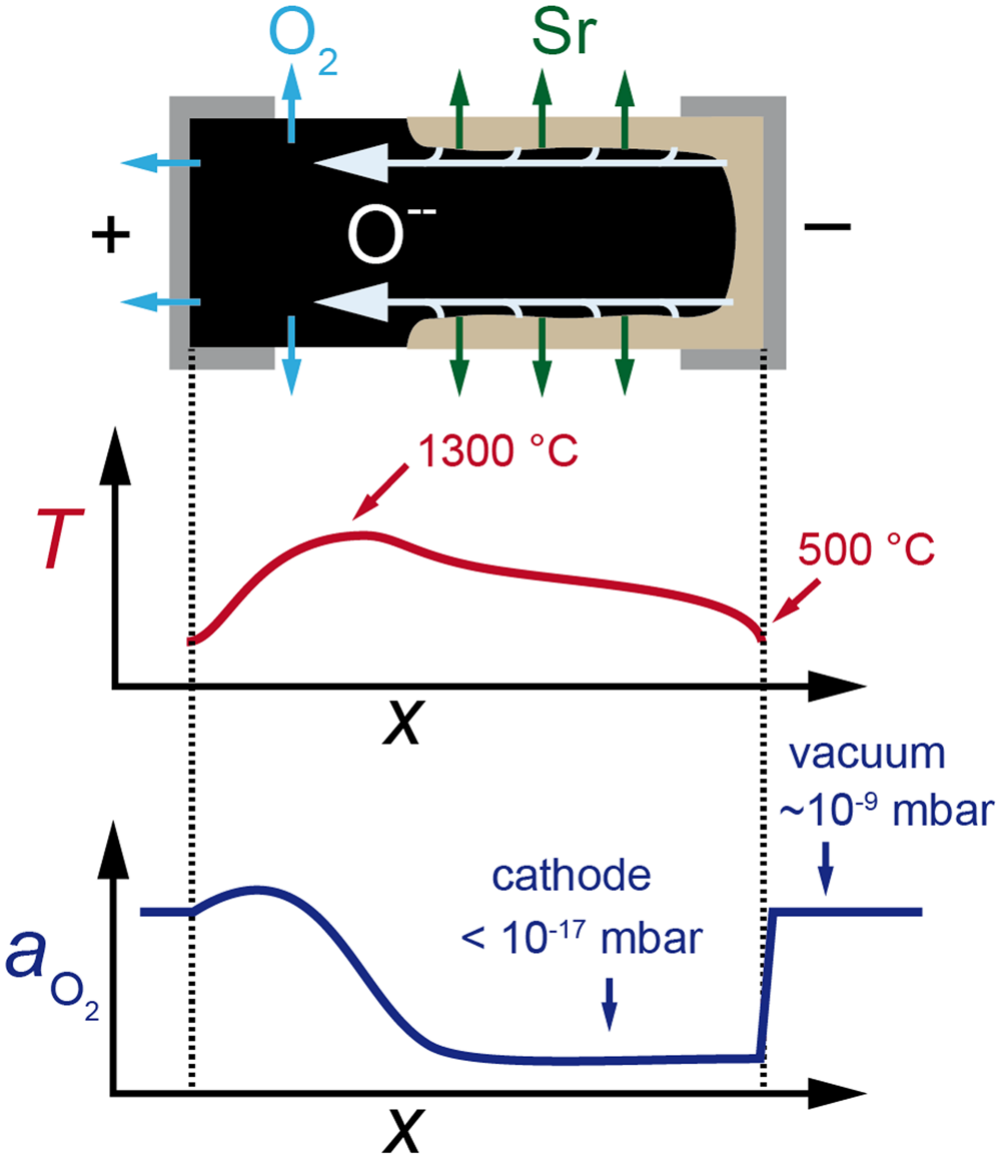
Schematic cross section diagram to qualitatively illustrate the proposed decomposition mechanism. Ionic flux, temperature (*T*) distribution and oxygen activity (*a*_O2_) during the electrodegradation process.

Having excluded these three effects as driving force of the decomposition, we now present our model of incongruent sublimation upon electrochemical polarization of titanates. When an in-plane electrical potential gradient is applied to the conducting oxide, the temperature is increased due to Joule heating. At elevated temperatures, the mobility of oxygen ions is high enough^24^ to allow a DC-voltage-driven migration of oxygen ions from the cathode towards the anode side. Such an oxygen movement is also known to occur in prototypical memristive devices^1,25^. Since we used porous Pt as electrodes, they can be regarded as a non-blocking electrode for oxygen transfer allowing for the release and uptake of oxygen to and from the vacuum. However, the cathode may act kinetically as a partially blocking electrode for oxygen uptake because it is surrounded by vacuum having low oxygen activity and not enough oxygen can be incorporated to balance the oxygen migration towards the anode^26^. We thus expect that the ternary oxide undergoes stoichiometry polarization. Consequentially, an in-plane gradient in the oxygen activity is built up in the course of time with a significantly reduced oxygen activity in the cathode region leading to incongruent sublimation from this region. The temperature distribution and the resulting oxygen activity is illustrated schematically in Fig. 5. Regarding the temperature profile in Fig. 5, it can be seen that the temperature at the electrode of the anode side is reduced since the clamps contacting the sample act as heat sink. Due to the lower temperature, the oxygen transport will be hindered and the maximum in oxygen activity is not located exactly at the anode but appears in the hotter part of the anode region. While this description gives a reasonable explanation of the decomposition process on the macroscale, its kinetics may additionally be influenced by defects and variations on the nanoscale (cf. Supplementary Information III). Our model of stoichiometry polarization is supported by optical analysis and electrical measurements of the bulk below the decomposed layer showing that also the bulk of the cathode region gets reduced while the bulk of the anode region is oxidized during the electrodegradation of SrTiO_3_:Nb (cf. Supplementary Information Id). We conclude that in the surface region of the cathode region, an extremely low oxygen activity is established. It has to be noted that this oxygen activity has to be much lower than that of the surrounding vacuum (The base oxygen partial pressure is approx. *p*_O2_ ≈ 10^−11^ mbar, which increases during the electrodegradation to max. 10^−8^ mbar due to oxygen release from the anode; see Supplementary Information Ia). As we have calculated and demonstrated recently by performing annealing experiments on SrTiO_3_ while the oxygen partial pressure was reduced by placing an oxygen getter such as Ti in close vicinity to the surface^14^, under such extremely low oxygen activities (*p*_O2_ < 10^−17^ mbar at 1000 °C), incongruent sublimation of the alkaline earth metal can be expected. Despite the high thermodynamic stability of SrTiO_3_, a decomposition via incongruent sublimation can take place via the vapour phase establishing above the surface of the oxide. This can be understood as follows. At first, SrTiO_3_ will volatize by partial dissociation to SrO(g) and TiO_2_(g). Since under strongly reducing conditions a subsequent dissociation of SrO(g) to Sr(g) and O(g) is thermodynamically much more favourable than a contrastable dissociation of TiO_2_(g), a high Sr partial pressure is established. In consequence, the loss of Sr into the vacuum is many orders of magnitude higher than the loss of Ti-containing species and the surface will decompose while TiO_x_ will remain forming a TiO_x_ surface layer. Based on the Ti-O phase diagram^27^, we must in principle consider that Ti suboxides and solid-state solutions of oxygen in titanium could form at the surface when the surface decomposes. This seems indeed to be the case. After cooling the partially disintegrated samples under vacuum conditions, various substoichiometric TiO_x_ compounds were found to exist in the decomposed surface layer as shown in Figs 2 and 3. In the course of time, the surface disintegration will proceed both towards the bulk of the oxide and towards the anode region following both the evolution of the stoichiometry polarization as well as the unidirectional shift of the electrical potential drop and temperature distribution over the slab towards the anode.

## Conclusion and Prospect

We have shown that ternary titanates can be decomposed via incongruent sublimation when the oxygen activity is lowered by the application of an electric field leading to oxygen excorporation and stoichiometry polarization. This way, a porous surface layer in the cathode region can be generated on a macroscopic scale with thicknesses of tens of micrometres. Regarding potential technical applications, we could imagine to exploit the observed electrochemically controlled decomposition in two ways. On the one hand, the generation of a mesoporous layer of defined TiO_x_ crystallites could be used for controlled surface engineering of ternary oxides by a technologically simple procedure. We demonstrated this process for doped and undoped SrTiO_3_, CaTiO_3_, and BaTiO_3_ indicating that a broad class of the ternary oxides can be used to obtain a self-supporting mesoporous TiO_x_ layer, e.g., for applications in the field of energy conversion, energy storage, filtering, and gas sensing, in which the application of titanium oxides, in particular of a porous structure, is under discussion^28–32^. As illustration of the different electronic properties of titanium suboxides we performed DFT calculation shown in Supplementary Information II. On the other hand, the related evaporation of alkaline earth metals in form of a molecular beam could allow for the growth of thin films as we demonstrate in Supplementary Information Ib. Above all, we have shown that it is possible to use an electrochemical method to achieve extremely low oxygen activities on free surfaces in vacuum without the need of sealing materials used typically in Hebb-Wagner experiments to generate blocking electrodes^33^. Under such low oxygen activities, which are much lower than the typical oxygen activities during thermal annealing, e.g. in H_2_ atmosphere, a decomposition via Sr evaporation takes place leading to the formation of TiO_x_ phases on top of the titanate. This could help to extend the SrO-TiO_2_ phase diagram with respect to the oxygen and strontium partial pressure. Furthermore, the interplay between the oxygen movement and the generation of local phase transformations provides a fundamental insight into the nature of processes during the electrodegradation of commonly used memristive materials and we expect that this will be highly relevant for the understanding of resistive switching phenomena. In prototypical oxide thin film memristive devices, owing to the class of valence change mechanism (VCM)^10,34–38^, it has been shown, e.g., for TiO_2_, that upon the initial application of an electrical gradient locally a secondary phase (here Ti_4_O_7_) can be formed acting as filament where the resistive switching takes place^39^. Similar effects have been found in SrTiO_3_ showing a local evolution of Sr-deficient filaments during electroforming^7,40^. Hence, we propose that the disintegration of SrTiO_3_ driven by the stoichiometry polarization described here may help to design dedicated methods to induce controlled local phase transformations, e.g., forming a switchable filamentary TiO_x_ region within a less conducting SrTiO_3_ matrix electrochemically which could improve the retention and reliability of memristive cells significantly.

## Methods

### Sample preparation

Epi-polished single crystals of SrTiO_3_ doped with 0.7 wt% Nb (Mateck), undoped SrTiO_3_ (Crystec), and BaTiO_3_ (FEE) obtained from Verneuil growth as well as ceramics of CaTiO_3_, prepared in-house by a conventional mixed-oxide process were investigated. Samples were fabricated by cutting a piece of the oxide material (7 mm × 3 mm × 0.5 mm) and contacting with pasted Pt electrodes. During electrodegradation, a voltage controlled by a Kepco BOP power source was applied under vacuum conditions (*p* < 10^−6^ mbar) leading to the presence of gradients of the electrical and chemical potential. The voltage applied was in the range of 5–15 V and the current in the range of 2–3 A. For all investigated oxides, comparable parameters were used. In order to distinguish between the influences of the electrical and thermal gradient, we chose a planar geometry making it convenient to monitor the temperature evolution during electrodegradation by an infrared camera.

### Characterization techniques

After electrodegradation, the chemical composition and electronic structure of the surface layer were investigated by X-ray photoelectron spectroscopy (XPS) with a PHI 5000 Versa Probe using monochromatized Al-K_α_ rays and a photoemission angle of 45°. Scanning electron microscopy (SEM) was performed on a Hitachi SU 8000 in combination with electron-dispersive X-ray spectroscopy (EDX) at an electron energy of 20 keV using an Oxford AZtec system to obtain maps of the element distribution. The crystallographic structure was investigated by electron back scatter diffractometry (EBSD) using a Hikari EBSD camera from EDAX-TSL attached to a JSM7000F FEG-SEM from JEOL, which was operated at a beam energy of 10 keV and a probe current of approx. 25 nA. The TEM imaging was performed at 300 kV accelerating voltage on a field emission gun FEI Titan 80–300 transmission electron microscope equipped with an imaging spherical aberration (*C*_S_) corrector. High-angle annular dark-field scanning transmission electron microscopy (HAADF-STEM) and electron energy loss spectroscopy were carried out at 300 kV with a field emission gun FEI Titan 80–300 transmission electron microscope equipped with a probe spherical aberration (*C*_S_) corrector and a post-column Gatan energy filter system. Energy-dispersive X-ray (EDX) element spectrum imaging was conducted at 200 kV with a field emission gun FEI Tecnai G2 F20 transmission electron microscope.

### Data availability

All data generated or analysed during this study are included in this published article (and its Supplementary Information files).

## Electronic supplementary material

Supplementary Information

Supplementary video
