# Supplementary material for: Electrically controlled transformation of memristive titanates into mesoporous titanium oxides via incongruent sublimation
Source: Sci Rep. 2018 Feb 28;8:3774. doi: 10.1038/s41598-018-22238-4 (PMC5830621; doi:10.1038/s41598-018-22238-4)
Supplement: Supplementary file 1 — Supplementary Information [file 41598_2018_22238_MOESM1_ESM.pdf]

Supplementary information

to

Electrically controlled transformation of memristive titanates into mesoporous titanium oxides via incongruent sublimation

C. Rodenbücher, P. Meuffels, G. Bihlmayer, W. Speier, H. Du,

A. Schwedt, U. Breuer, C.-L. Jia, J. Mayer, R. Waser, K. Szot

I. DETAILS OF THE DECOMPOSTITON PROCESS

Ia. TIME DEPENDENCE OF ELECTRODEGRADATION

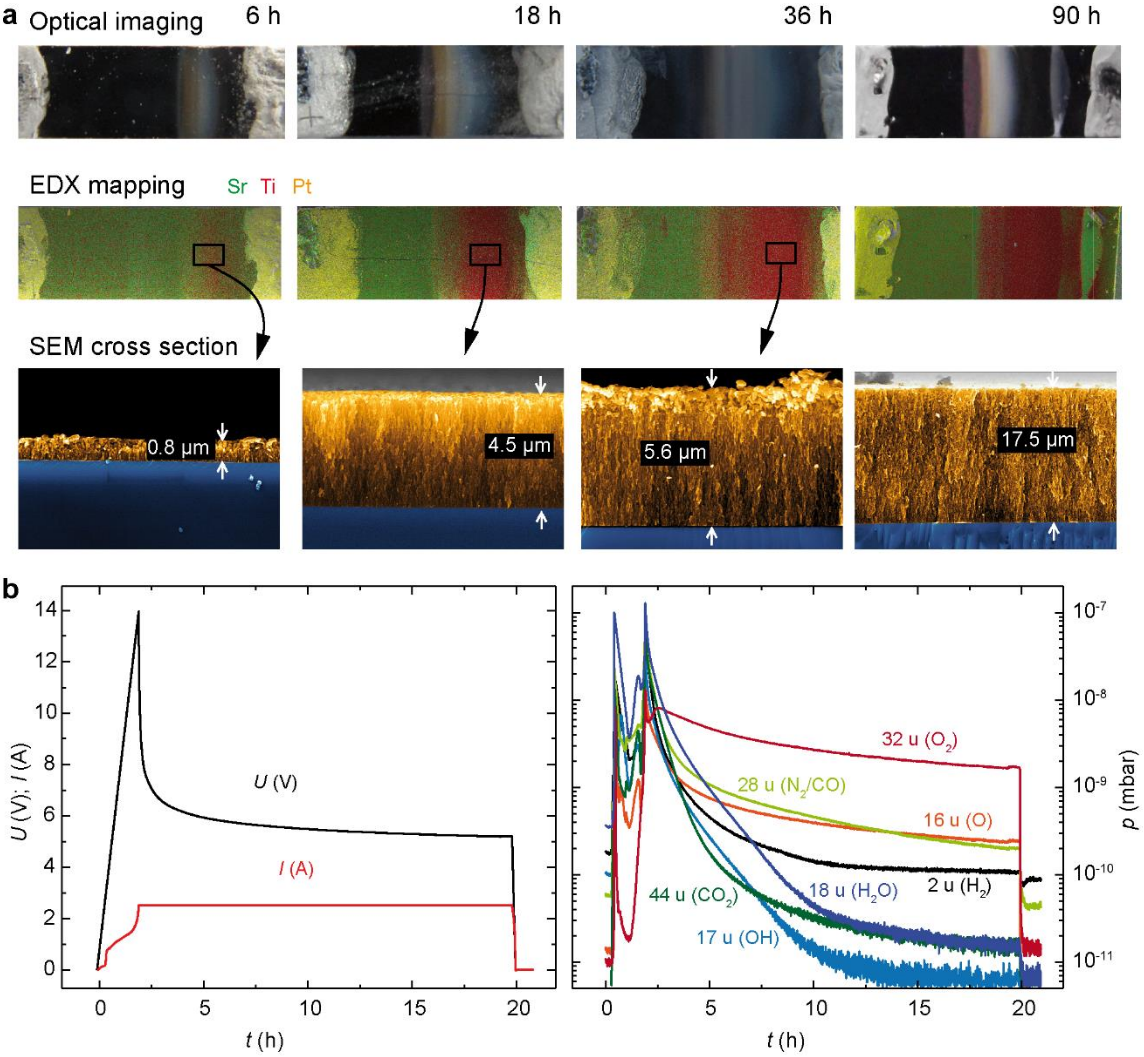

Figure S1. Analysis of electrochemical decomposed $SrTiO_3$:Nb crystals as function of degradation time. (a) Optical images, EDX maps and SEM cross sections of samples degraded for different times. (b) Voltage, current and partial pressures in the vacuum chamber recorded during degradation for 20 h.

To analyse the time dependence of the decomposition we conducted a series of measurements on $SrTiO_3$:Nb crystals electrodegraded for different times from several hours up to several days. All samples were treated in a vacuum below 10^{-6} mbar, and a DC voltage with current compliance of 2.2 A was applied, corresponding to a voltage of max. 14 V. While at times shorter than 6 h no significant changes in the crystal were visible, after prolonged treatment, the original black colour of the crystal changed to a striped pattern of reddish to greyish colour as shown in the optical top-view images of Fig. S1a. Using EDX mapping, we correlated the colour change to the evolution of a Ti-rich region, in which almost no Sr was present, showing that a fundamental disintegration from $SrTiO_3$ into TiO_x had taken place locally in the cathode region. The thickness of this TiO_x region was determined by performing scanning electron microscopy (SEM) cross section imaging after cleaving the sample. Starting from decomposed layer of only a few hundred nanometres the layer thicknesses increased up to tens of micrometres showing that a continuous decomposition and release of Sr takes place at the border between layer and bulk slowly eating away the crystal at the cathode side. When using undoped $SrTiO_3$ (self-doped by thermal reduction prior to electrodegradation), this process is even faster and we managed

to create nanoporous layers with thicknesses up to 50 µm within a few hours most probably due to the higher ionic conductivity in the undoped material.

In Fig. S1b, the evolution of the voltage and the composition of the atmosphere inside the vacuum chamber during electrodegradation is shown. When ramping up the voltage, the power increased leading to Joule heating desorbing molecules from sample and sample holder associated with significant effusion of carbon oxides and water. After this first burst, the power decreased again since the current compliance was reached and the voltage decreased due to the increase in conductance of the sample during the degradation process. Simultaneously, the partial pressure of carbon oxides, hydrogen oxides, and hydrogen decreased but the oxygen partial pressure stayed relatively high (above 10^{-9} mbar) indicating that a huge amount of oxygen was released from the sample upon electrochemical polarization. After 20 h, the voltage was switched off and the oxygen partial pressure immediately dropped below $2 \cdot 10^{-11}$ mbar while hydrogen became the major constituent of the atmosphere in the chamber typical for UHV conditions.

Ib. STRONTIUM EVAPORATION

Having seen that decomposition in the surface region took place leaving behind a TiO_x-rich layer, we now address the evolution of the corresponding Sr component. While we could not find any evidence of a Sr segregation in the bulk or at the anode, condensation of Sr-rich material was observed on nearby colder parts in the vacuum chamber showing that Sr indeed evaporated during the electrodegradation runs. This was confirmed by mass spectrometry (here the degradation was performed at 3 A in order to clearly identify the Sr signal). As described in the main text, we assume that incongruent sublimation of Sr occurs. To support this idea, we compared the measured Sr sublimation during electrodegradation with the thermally induced sublimation from $SrTiO_3$ powder mixed with the oxygen getter Ti[1]. For reference, we additionally measured a mass spectrum during thermal annealing of pure $SrTiO_3$ powder. While pure $SrTiO_3$ did not show any Sr evaporation above the detection limit up to temperatures of 1500 °C, the addition of an oxygen getter generated a significant Sr signal already at temperatures below 1000 °C. DC polarization resulted in a comparable Sr evaporation (Fig. S2a). In all cases, only the evaporation of metallic Sr was recorded while the signal of other metal-based species in particular SrO with a mass of 104 u was not found.

In order to locate the source region of the evaporating species during DC polarization, we used a masked deposition sheet mounted directly at a distance of 3 mm above the sample in the vacuum chamber as illustrated in Fig. S2b. As a mask we used a boron nitride sheet in which a row of holes with diameters of 30 µm was cut and which was covered with Pt foil serving as deposition sheet. After electrodegradation, we analysed the deposited dots on the Pt sheet by SEM/EDX. As shown in Fig. S2b, EDX line scans along the line of dots on sheets mounted above the decomposing sample for different times corresponding show that the maximum Sr intensity was found in the dots positioned above the cathode region. This indicates that a molecular beam of Sr evaporated directly from the decomposing cathode region. To demonstrate in a proof of principle approach that the observed Sr evaporation allows for the deposition of Sr-rich layers via molecular beam epitaxy we performed a test by using the decomposing sample as target and a Si crystal as substrate mounted at two different distances above the cathode region of the $SrTiO_3$:Nb crystal. After the electrodegradation was conducted for 8 h which is enough to start the Sr evaporation according to Fig. 3, a shutter between target and substrate was opened while the DC voltage was permanently applied to the $SrTiO_3$. After 1 h of deposition, the layers on the epi-polished Si sample were analysed by AFM. It can be seen in Fig. S2c that crystallites with heights up to 7 nm had evolved. When the distance between target and sample were reduced from 2 cm to 1 cm the height of the crystallites even reached 25 nm and fully covered the Si surface. After prolonged deposition (18 h), macroscopic self-supporting flakes with thicknesses of several micrometres could be generated as illustrated in the cross-sectional SEM image in Fig. S2d. To analyse the detailed chemical composition and electronic structure of the deposited material, we performed in situ XPS measurements

during the electrodegradation runs under our typical vacuum conditions (~10^{-7} mbar). To this end, we constructed a dedicated sample holder (Fig. S2e) equipped with a shield preventing the Sr from evaporating into the XPS analyser. A typical XPS spectrum of the material deposited on the Pt sheet is shown in Fig. S2f. It can be seen that most of the material consisted of SrO with a very small amount of TiO_x. However, the overall Sr/O ratio was larger than 1 indicating the existence of metallic Sr. The Sr3d core line consisted of a main SrO doublet and a smaller doublet shifted by approx. 1 eV to higher binding energy. Although there are several opinions in the literature concerning the position of the Sr3d line [2], there are reports that the binding energy of metallic Sr is higher than that of SrO and hence the second doublet could be attributed to Sr. This shows that when metallic Sr condenses on a colder substrate, the main part of the condensing material reacts with residual oxygen gas in the surrounding vacuum to form SrO due to the high oxygen affinity of Sr. While we performed the experiments at a base pressure of approx. $3 \cdot 10^{-8}$ mbar, an improvement and precise control of the vacuum conditions could even allow for tailoring the properties of the deposited material via adjusting the Sr/O ratio.

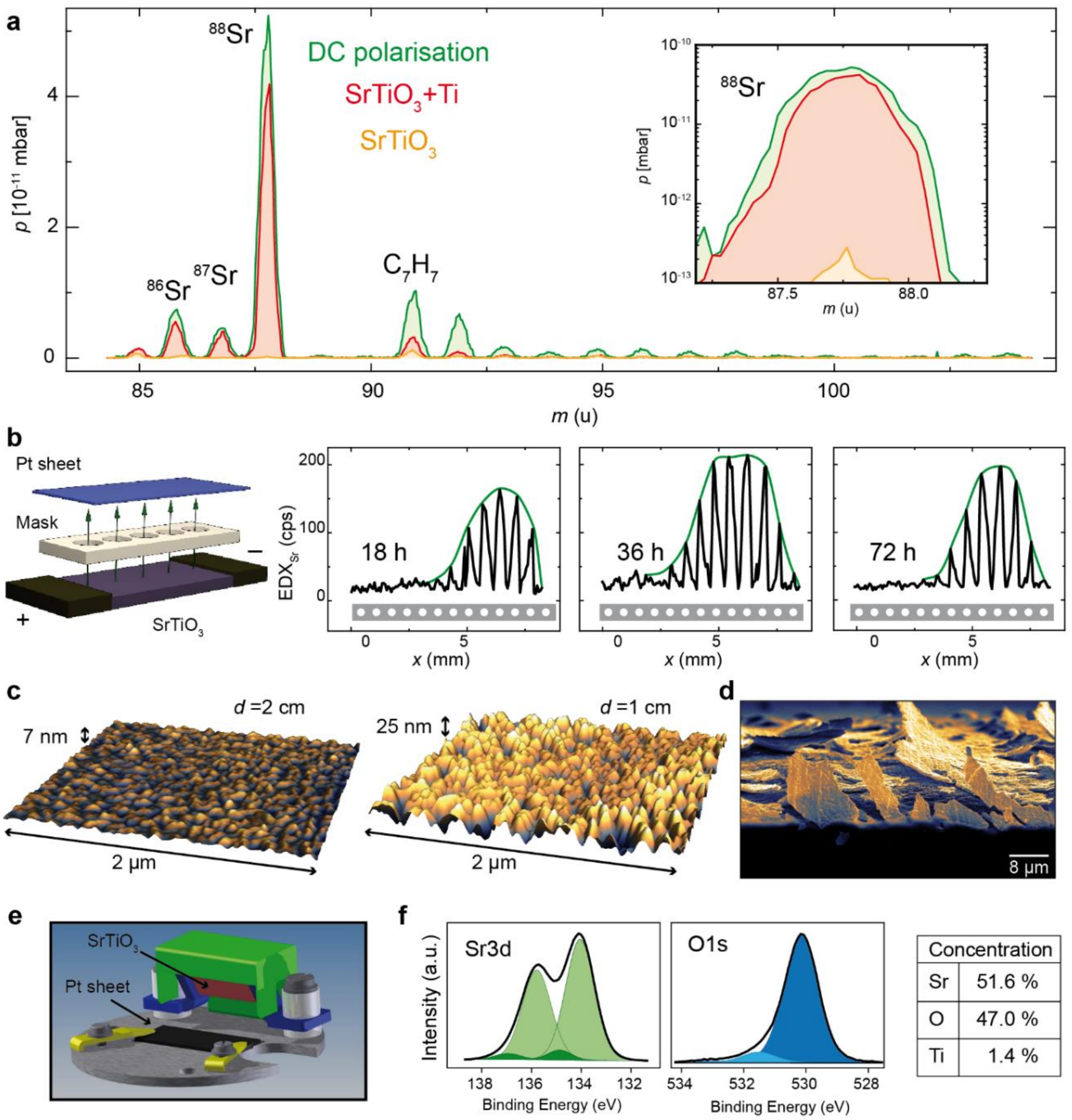

Figure S2. Sr evaporation. (a) Mass spectrometry during DC polarization compared to thermal annealing of a $SrTiO_3$/Ti mixture and pure $SrTiO_3$. (b) Outline of the masked deposition sheet and EDX analysis of the Sr content in the evaporated dots on the sheet. (The position of the holes is marked by white circles). (c) AFM analysis of SrO_y layers deposited on Si in a distance of 2 cm (left) and 1cm (right) above the $SrTiO_3$ sample. (d) Cross-sectional SEM image of SrO_y after long-time (12 h) electrodegradation. (e) Outline of the XPS sample holder. (f) XPS in situ analysis of the evaporated material.

Ic. EXCLUSION OF THE SORET EFFECT

The decomposition of the sample after electrodegradation raises the question whether the electrical potential gradient or the temperature gradient induced by the asymmetric potential drop is the main driving force. It is known that also a temperature gradient can cause ionic movements known as the Soret effect. In order to answer this question we applied DC voltage to one sample and AC voltage to another. The latter sample was cut in an asymmetric shape to induce a comparable temperature gradient at approx. 1500 °C on the hot side and approx. 600 °C on the cold side. The comparison of the chemical composition of the two samples after treatment for 15 h shows that the presence of a temperature gradient alone did not lead to decomposition. This is supported by XPS analysis. While in the DC case the Ti valences changed on the relatively cold cathode side due to the evolution of substoichiometric titanium oxides, in the AC case additional Ti valences +3 and +2 were observed only in the hottest region. Hence, in the latter case, valence change can be attributed to thermal reduction, which also involves a decrease in the Sr content of the very surface of about 30% presumably due to a movement of SrO towards the bulk as revealed by SIMS depth profiling of reduced $SrTiO_3$ crystals[3]. Additionally, we can derive from the effusion analysis of pure $SrTiO_3$ powder (Fig. 1) that $SrTiO_3$ is stable even at high temperatures under standard vacuum conditions ($p_{O2} \approx 10^{-11}$ mbar). In summary, we can conclude from this comparison between electrodegradation and thermal reduction that neither the Soret effect nor preferential thermal evaporation under standard vacuum conditions from the perovskite plays a significant role during the transformation of the surface layer but that the combination of high temperature and extremely low oxygen activity is needed to enable incongruent evaporation.

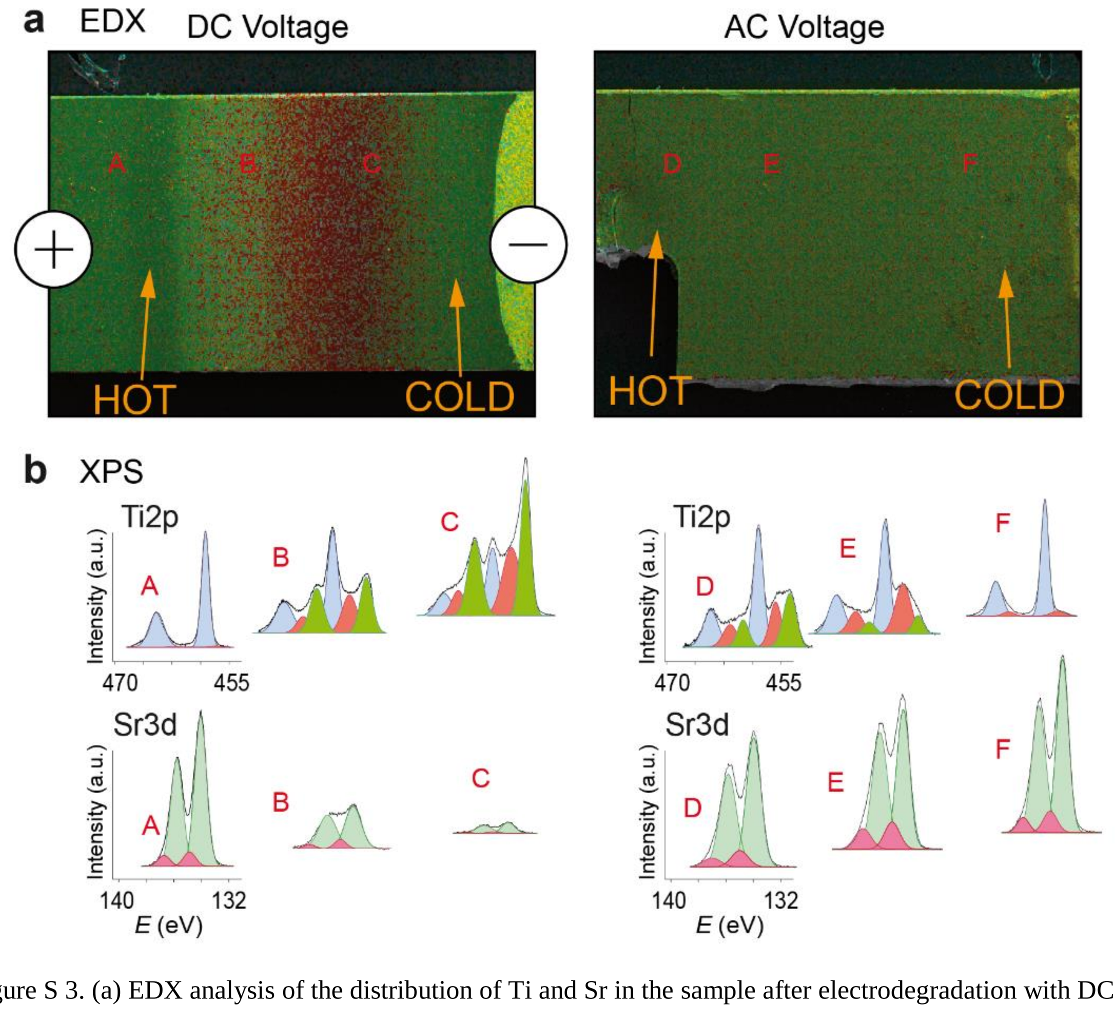

Figure S 3. (a) EDX analysis of the distribution of Ti and Sr in the sample after electrodegradation with DC voltage (left) and after treatment of an asymmetric sample with AC voltage (right). (b) Corresponding core line spectra of Ti2p and Sr3d measured by XPS.

Id. STOICHIOMETRY POLARIZATION

As stated in the main text, we suppose that the underlying mechanism of surface decomposition is a stoichiometry polarization upon application of a DC voltage. To illustrate this effect, we analysed the bulk of the crystal after electrodegradation. We polished a decomposed 0.5 mm thick $SrTiO_3$:Nb crystal from both sides in order to remove the porous TiO_x layer in the cathode region and analysed the sample having a remaining thickness of approx. 0.3 mm with optical microscopy in transmission-light mode. As shown in Fig. S4, it can be seen that the bulk of the cathode region had a dark black colour. The anode region instead, was translucent with a blue/violet colour. The magnification of the transition region depicts that the transition between both regions was not sharp but showed a certain degree of inhomogeneity on the microscale. The optical inspection indicates that the cathode region had a much higher absorbance than the anode region which supports the model of stoichiometry polarization related to an oxygen migration from cathode to anode. Additionally, an oxygen excorporation took place since the crystal has to be regarded as open system during electrodegradation under vacuum conditions. In consequence, the cathode region was reduced resulting in an increase of oxygen vacancy concentration and *d*-electrons, leading to a higher light absorbance while the anode region got oxidized and the concentration of *d*-electrons was reduced. To confirm this, electrical four-point measurements were performed. The polished bulk was cut in five slices as illustrated by the red dotted lines in Fig. S4b and each slice (size 3 mm x 2 mm 0.3 mm) was contacted with four sputtered Pt electrodes. Then the resistance was determined by measuring the potential drop between the inner electrodes while a current of 30 mA was flowing through the slice. As illustrated in Fig. S4b, bottom, the resistance at the cathode side was indeed reduced below the value of as-received Nb-doped $SrTiO_3$ (green dashed line) while the resistance at the anode side was indeed increased significantly, which supports the described model of stoichiometry polarization.

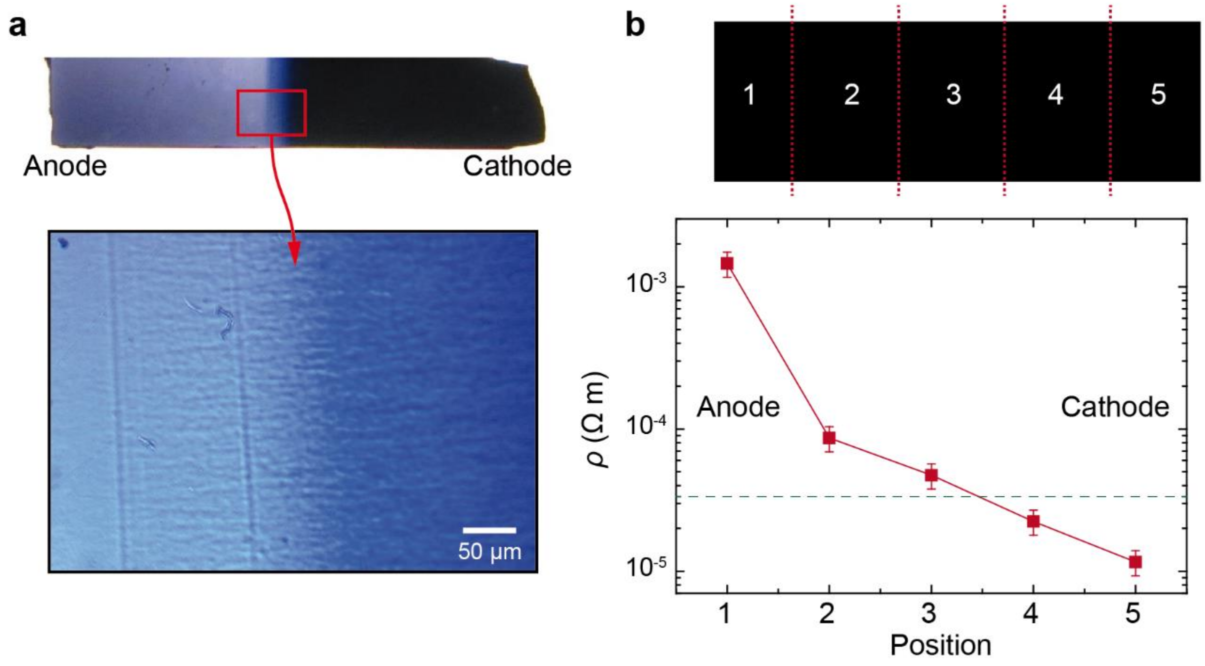

Figure S 4. (a) Optical microscopy in transmission light mode of an electrodegraded crystal after polishing away both surfaces. (b) Measurement of bulk conductivity on slabs of the polished crystals obtained by four-point method at room temperature.

Ie. DETAILS OF EBSD INVESTIGATION

The detailed SEM analysis in top view of the $SrTiO_3$:Nb after electrodegradation confirmed that the surface of the anode side had not changed significantly and was still smooth. In the Ti-rich region however, crystallites of different shapes were visible. While in the intermediate region crystallites had grown on top of the $SrTiO_3$ surface, in the centre of the TiO_x region a porous structure had evolved where the size of the grains and pores increased towards the cathode. The crystallographic structure of these grains was analysed by electron backscatter diffraction as shown in Fig. 2c. Here, in Fig. S5, the detailed Kikuchi pattern with the assignment of the crystallographic directions are shown. The patterns were obtained without further preparation of the surface. The pattern measured close to the anode can be described as a perovskite phase revealing that the crystal did not change very much in this region despite the high temperatures. In the Ti-rich part, the pattern recorded by EBSD in different grains can no longer be described as perovskite, but the cubic TiO (NIST 47537 as provided in the reference database by EDAX-TSL) and trigonal Ti_3O (NIST 47547) were identified in agreement with the XPS results.

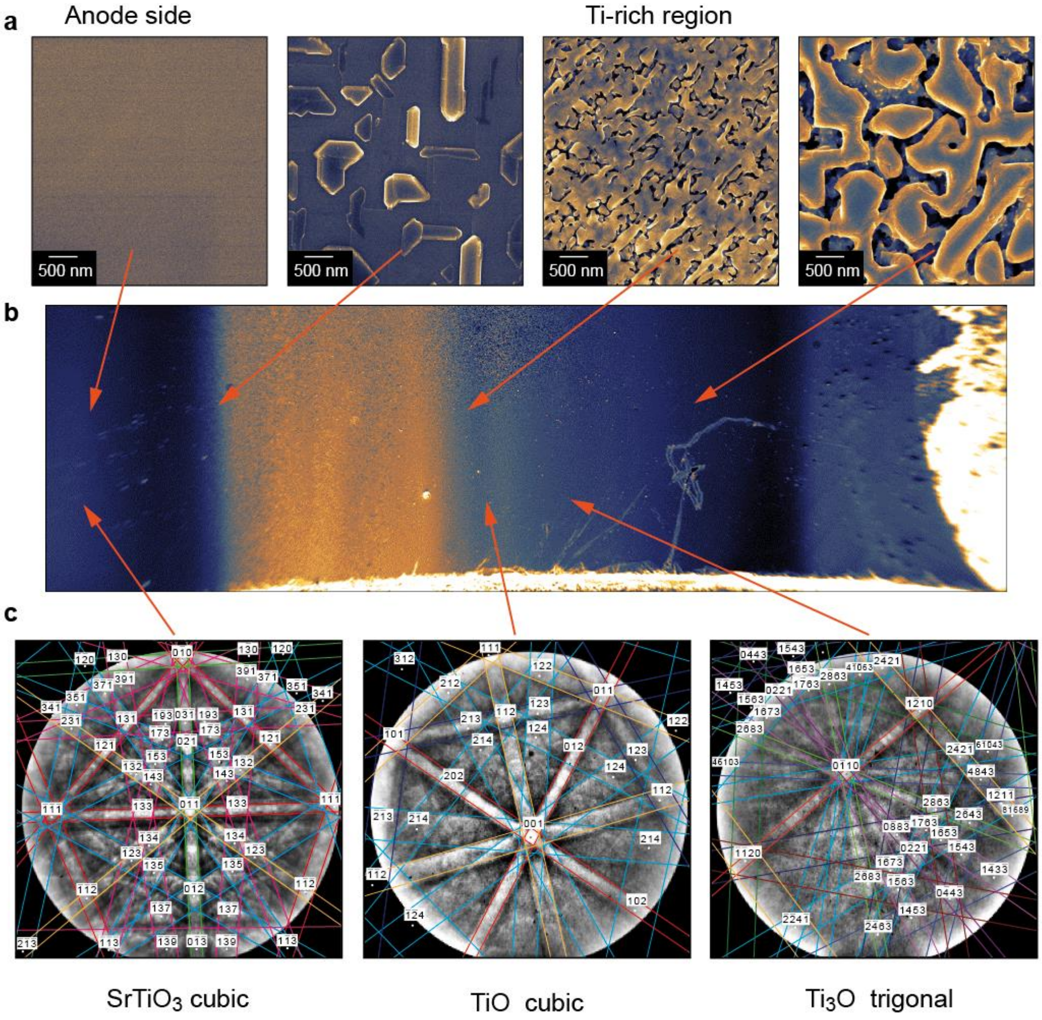

Figure S5. (a) SEM images obtained at different positions between anode and cathode as marked in the (b) SEM overview image. (c) Corresponding EBSD patterns and indexing.

If. DECOMPOSITION UNDER ARGON/HYDROGEN ATMOSPHERE

The electrodegradation experiments presented in the main text were conducted under vacuum conditions at a total pressure below 10^{-6} mbar maintained by a turbomolecular pump. However, in terms of electrochemistry these conditions are thermodynamically not well defined in particular since the local oxygen partial pressure does not only depend on the total pressure but also e.g. on the materials used inside the chamber[1]. In order to obtain comparability to electrochemical measurement methods, we conducted an electrodegradation experiment in a continuous flow of Ar mixed with 4 % H_2 under atmospheric pressure establishing reducing conditions in the surrounding. The degradation was performed inside a quartz tube which was hold at 900 °C by an external heater. We applied comparable electrical gradients (U_{max} = 10 V, I_{cc} = 2.2 A) via pasted Pt electrodes as in the vacuum experiments. After a degradation time of 24 h, the sample was analysed by means of SEM and EDX as presented in Figure S6. It can be seen that a change in stoichiometry occurred close to the cathode but next to Ti-rich areas (red colour) also Sr-rich areas (green colour) are present on the surface. SEM magnification of the Sr-rich surface of the cathode region reveals the presence of flower-like crystallites presumably consisting of SrO. After mechanical cleaving, the cross section of the cathode region was analysed by SEM/EDX. It can be seen that a Sr-depleted TiO_x layer has evolved as in the vacuum experiments but is covered by an additional layer of SrO_y. This finding supports our model that the decomposition is caused by incongruent sublimation at low oxygen partial pressure. Since the atmosphere is highly oxygen deficient in Ar/H_2 as well as in vacuum, the stoichiometry of the surface cannot be maintained when the sample is polarized resulting in an extremely low oxygen activity at the surface leading to incongruent sublimation of Sr. While in vacuum, the evaporated Sr atoms can freely move and condense on the walls of the chamber, this sublimation is suppressed in Ar/H_2 atmosphere due to the smaller mean free path. Hence, the Sr atoms are bounced back to the surface of the crystal and are oxidized there at least when the sample is transferred ex situ to the electron microscope. Interestingly, such a sequence of TiO_x covered with SrO_y has also been found below the top electrode of prototypical memristive $SrTiO_3$ devices after electroforming[4].

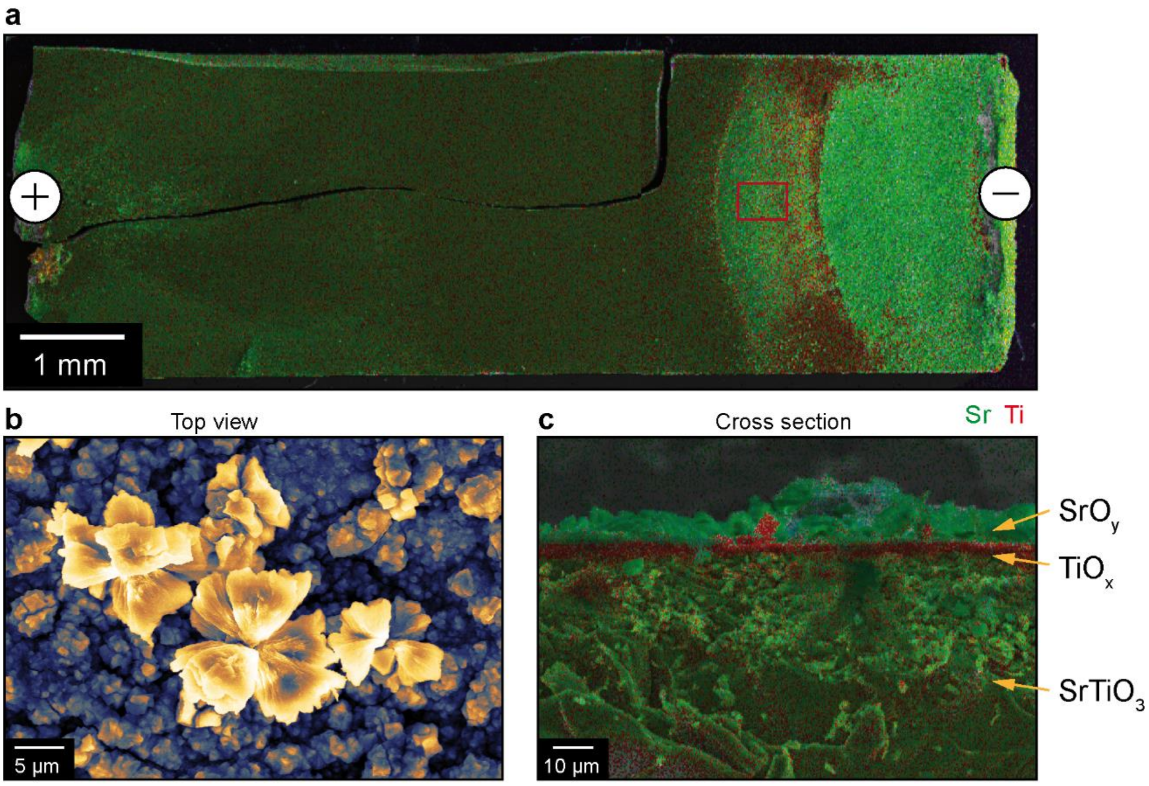

Figure S6. (a) SEM/EDX top view of the $SrTiO_3$:Nb crystal degraded under Ar/H_2 atmosphere. (b) SEM top view of the surface close to the cathode showing SrO_y crystallites. (c) SEM/EDX cross section of the cathode region obtained after mechanical cleaving.

IIa. POINT DEFECT CHEMISTRY

In order to explain that doped and nominally undoped $SrTiO_3$ (which can be assumed to be acceptor-doped due to impurities and/or Sr vacancies introduced as Schottky defects at crystal growth temperatures) exhibited comparable behaviour under electrodegradation, we suggest the following: In typical regimes of temperatures T and oxygen partial pressures p_{O2}, donor- and acceptor-doped $SrTiO_3$ show very different behaviour[5]. The first is a good electron conductor due to the compensation of positively charged donor centres by electrons, while the second is a poor electronic conductor which shows some additional ionic conductivity due to the compensation of the negatively ionized acceptors by mobile oxygen vacancies.

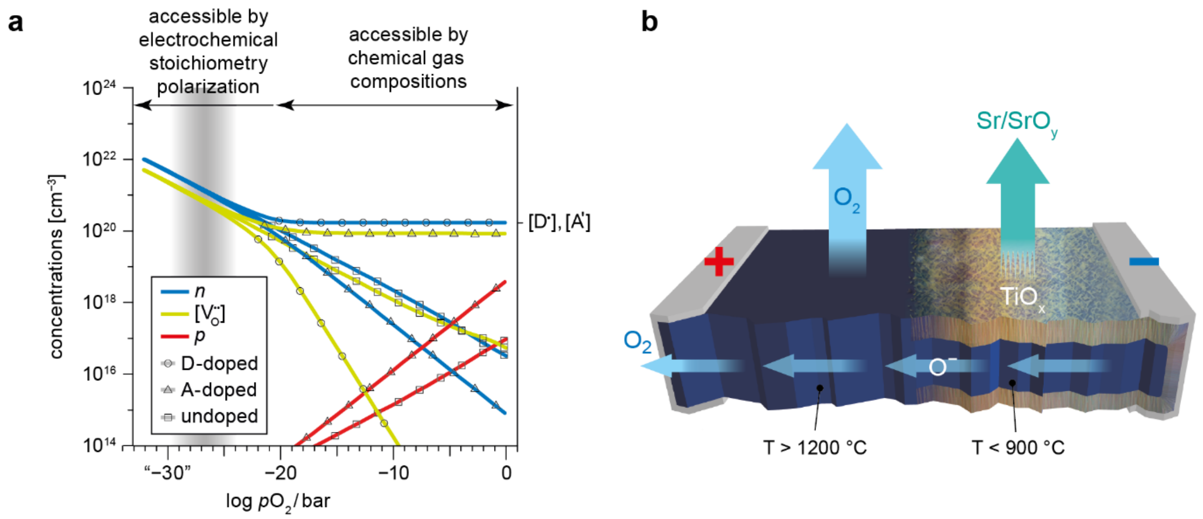

Figure S7. (a) Illustration of the equilibrium concentration of oxygen vacancies ($[V_O^{\cdot\cdot}]$, yellow) and electrons (n, blue) as a function of the oxygen partial pressure (log pO_2) for 1 at% donor-doped ($[D^{\cdot}]$, circles), undoped (squares), and 1at% acceptor-doped ([A´], triangles) $SrTiO_3$ at 1500 K, using the Kroeger-Vink notation [5]. The sketch shows that for very reducing conditions, in particular conditions which can be reached by electrochemical polarization, the system approaches the same high concentrations of electrons and oxygen vacancies, independent of the cation dopant concentrations. (b) Schematic illustration of the sample geometry and the assumed ionic movements. Graphics by T. Pössinger.

Therefore, it is intriguing that differently doped $SrTiO_3$ (as well as even other perovskites, see Fig. 1) show the reported effects of stoichiometry polarization and phase formation in a very similar fashion. The extended Brouwer diagram Fig. S7 may offer an explanation. The diagram was calculated from mass action data presented by Moos and Härdtl[6] without considering differently charged oxygen vacancies, vacancy clustering, or correlation effects, for simplicity. It can be seen in Fig. S7 that the concentrations at intermediate and high pressures, pO_2, are significantly different but that they converge in the low oxygen partial pressure regime. In our experiments, a high-temperature UHV reduction of $SrTiO_3$ shifts the defect situation towards the left side in the Brouwer diagram. The electrochemically induced stoichiometry polarization due to the applied voltage drives the systems much further to the left. Using this technique, extremely high oxygen vacancy concentrations can be reached, much higher than those accessible by reducing gases. The authors are aware of the fact that the point defect chemistry picture strictly only holds for dilute defect concentrations so that the diagram in Fig. S7 does not reflect quantitative situations. Nevertheless, it is a valuable tool for indicating trends. This trend clearly shows that the oxygen activity in the cathode region was reduced so extremely due to electrochemical polarization that the defect situation becomes the same, irrespective of the nominal doping level. Under such reducing conditions, additional processes set in, which lead to a depletion of Sr (or Ca, Ba in case of $CaTiO_3$ and $BaTiO_3$) starting from the surface and resulting in the evolution of a TiO_x layer of comparable size and shape for all materials studied here. It is interesting that the $SrTiO_3$ underneath the

near-surface TiO_x layer retains its perovskite crystal structure despite the extremely high oxygen vacancy concentration. This reflects the high stability of the perovskite lattice (although distorted) and is consistent with an earlier report on a $SrTiO_3$ with an unusually high O deficiency which was synthesized from SrO and TiO_x and, as expected, exhibits a long-range ordering of the oxygen vacancies.[7]

IIb. ELECTRONIC STRUCTURE OF TITANIUM SUBOXIDES

Using X-ray photoelectron spectroscopy (XPS), we determined the chemical composition and electronic structure in the region between the anode and cathode that could be clearly identified by the change in colour. The Sr/Ti and Ti/O ratio extracted from the measurements of the core line spectra at different positions is shown in Fig. S8a. In agreement with the EDX maps in Fig. 1, on the anode side, typical spectra of $SrTiO_3$ were obtained with a Ti valence of +4 proving that the crystal did not change very much in this area. Towards the TiO_x region on the cathode side, a distinct depletion of Sr was found and the Ti valences +3 and +2 dominated showing that the valences had not only changed during the polarization[8] but that a remanent transformation into new phases had taken place. The Ti/O ratio increased simultaneously over a distance of a few millimetres from values of 0.3 ($SrTiO_3$) to values of 3 corresponding to Ti_3O in the decomposed region, which is in agreement with the EBSD results. In between the two extrema, the Ti/O ratio takes various intermediate values indicating that a phase transformation into TiO and a variety of Ti_nO_{2n-1} suboxides, which are known to exist in the Ti-O system[9], had occurred.

To illustrate the variability of the electronic structure of those Ti oxides generated during electrodegradation in the surface region and their potential for resistive switching behaviour, we performed density functional theory (DFT) calculations in the generalized gradient approximation[10]. We calculated the density of states (DOS) of TiO_x (x = 2, 9/5, 3/2, and 1) in the paramagnetic phase in the generalized gradient approximation (see Fig. S8b). To account for correlation effects in the TiO_x we used the DFT+U model with U_d = 5.5 eV for x=2 and U_d = 4.5 eV for x=9/5 and 3/2. Since we are interested in the properties at high temperatures, no magnetic effects were taken explicitly into account. In this study, the full-potential linearized augmented planewave method as implemented in the Fleur code was used (for a program description see http://www.flapw.de). In all cases, the experimental lattice structures of the ground state phases were assumed. These calculations do not capture all the complex charge and orbital order effects at low temperatures, nor all the subtle correlation effects in some of these compounds. The general trend of the conductive behaviour at higher temperatures should, however, be correctly reflected. In this approximation, rutile TiO_2 is an insulator with 2.0 eV bandgap (as usual, within DFT the gap is underestimated as compared to the experiment), while in Ti_5O_9 metallic Ti states from the conduction band enter the gap and cross the Fermi level. We can regard this compound as a composite made out of corundum (Ti_2O_3) and rutile and write it $Ti_2O_3{\cdot}(TiO_2)_3$. The metallic states can be traced back to the Ti atoms with a local corundum structure (blue-shaded DOS). Although there are states at the Fermi energy forming conductive (121) planes throughout the crystal, the total conductivity of the phase is still rather low. At lower temperatures, when magnetic and orbital ordering sets in, even a small band gap can be formed[11]. With increasing oxygen deficiency all Ti are finally in oxidation state +3 in the corundum structure. Ti_2O_3 is a Mott-Hubbard insulator and the DOS shows a small gap of 0.2 eV, a value that is quite comparable to a recent study using the screened exchange functional[12]. Further reduction leads to TiO (here, we show the α-phase, but similar results are also obtained for the cubic structure[13]) where metallic Ti states are present at the Fermi level. These four examples illustrate the rather complex dependence of the electronic properties of TiO_x phases with the oxygen content (x), showing an overall increase of conductivity with decreasing x, but with a distinct minimum around x=3/2 due to correlation effects. This illustrates that electrodegradation opens up the

opportunity to generate phases with significantly different electronic structure, which may be highly relevant for the evolution of switchable filaments in resistive switching memory cells.

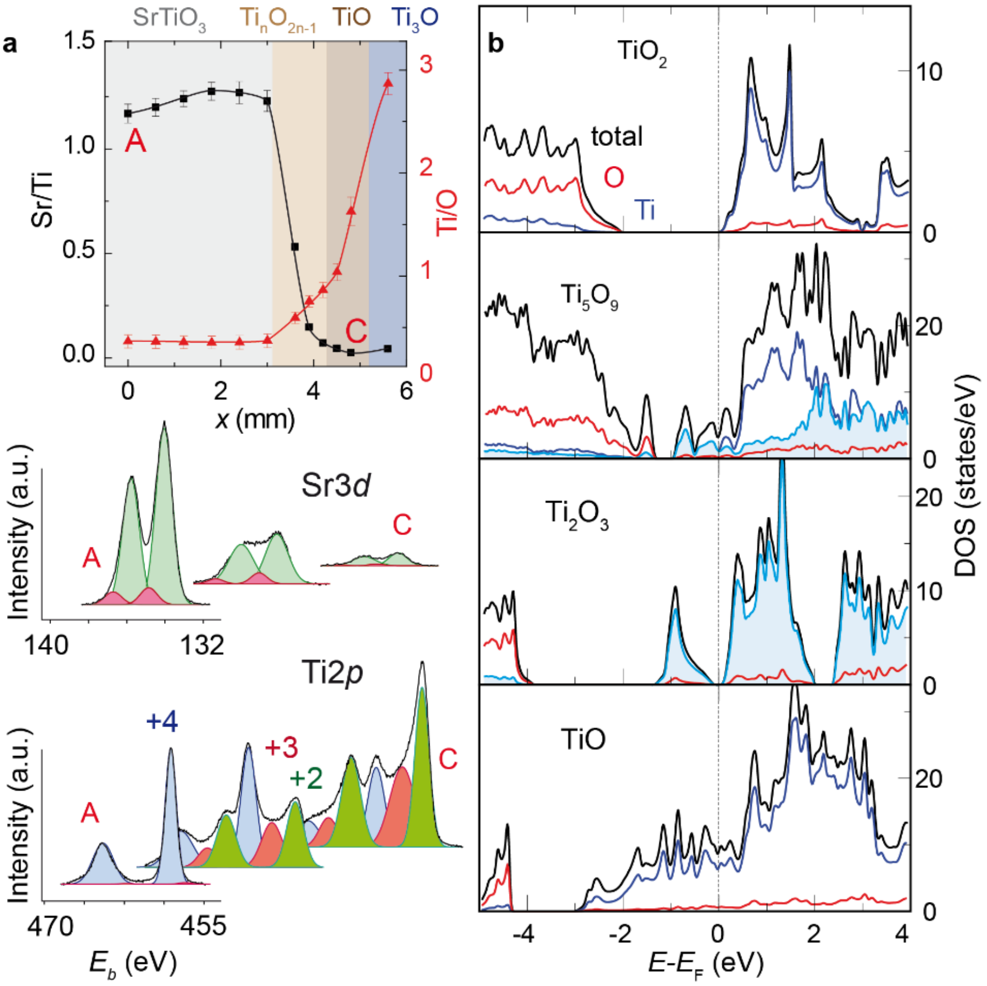

Figure S8. Electronic structure. (a) XPS measurement of the chemical composition (top) and electronic structure (bottom) between the anode (A) and cathode (C) region. (b) Total (black) and local (colour) DOS of four selected titanium oxides phases in the paramagnetic state. The oxygen DOS is shown in red, the titanium DOS in blue. In Ti_7O_9 and Ti_2O_3 the DOS from the Ti^{3+} ions is indicated by the blue shading

IIIa. INFLUENCE OF CRYSTAL GEOMETRY

The porous TiO_x surface layer consisted of nanoscale crystallites with a characteristic geometric shape aligned along the crystal axis. As shown in the SEM images Fig. S9 obtained at different positions in the cathode region, the rims of the crystallites are orientated either along the <100> axes of the original $SrTiO_3$ crystal or diagonal along <110>. This shows that there is a close relation between the evolving crystallites and the supporting cubic crystal structure which could be mediated by extended defects such as dislocations. In the Verneuil-grown $SrTiO_3$ crystals we used, dislocations are often agglomerated in bundles forming linear structures that are also aligned along the crystal axes as revealed by optical microscopy after marking the exits of dislocations by etch pits using hydrofluoric acid. Hence, we assume that the presence of dislocations influences the process of decomposition by incongruent sublimation e.g. by serving as fast diffusion paths for Sr.

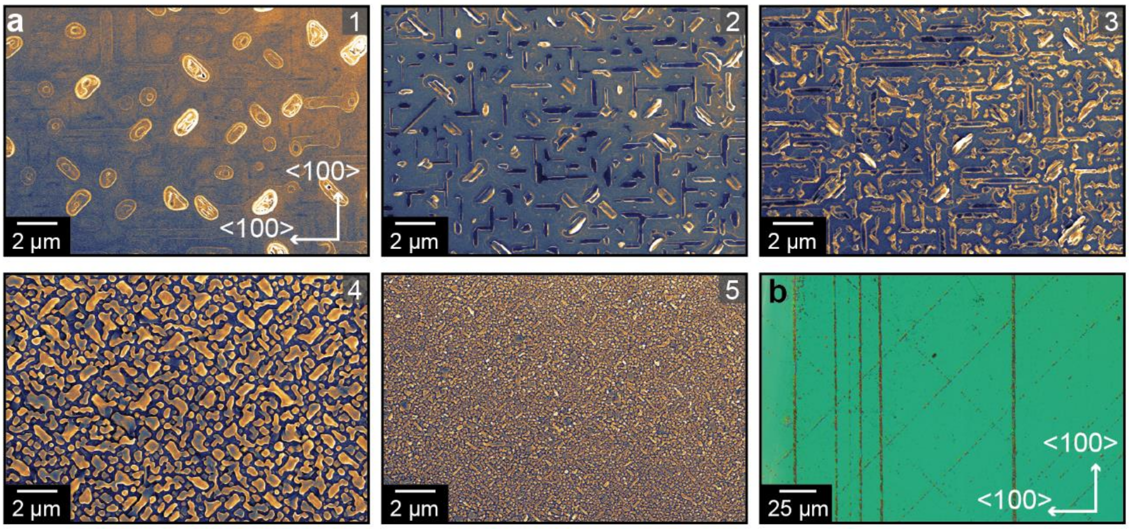

Figure S9. (a) SEM image of the crystallites formed during electrodegradation of undoped $SrTiO_3$. The sequence of images was obtained at different positions between anode (1) to cathode (5). (b) Optical microscopy of the arrangement of dislocations in a $SrTiO_3$ crystal marked by chemical etching.

IIIb. DIFFERENCES BETWEEN DOPED AND UNDOPED $SrTiO_3$

As shown in the main manuscript, the effect of decomposition via Sr sublimation upon electrochemical polarization was found for doped as well as for undoped $SrTiO_3$. Within the point defect chemistry model this can be understood taking into account that for low oxygen activities the concentration of vacancies is similar as shown in Fig. S7. Although this trend holds true in general, there are subtle differences regarding in particular the kinetics of surface transformations between Nb-doped $SrTiO_3$ and undoped $SrTiO_3$, which was self-doped by oxygen vacancies via thermal reduction. This can be seen at first regarding the temperature distribution shown in Fig. S10. In $SrTiO_3$:Nb, an asymmetric temperature distribution evolves after several hours of polarization (cf. Fig. 2) with a large area of the anode region showing elevated temperatures compared to the cathode. In undoped $SrTiO_3$ instead, an asymmetric temperature profile is established within a few minutes at comparable power and the region of elevated temperature is concentrated at the anode itself (Fig. S10a). In consequence, the surface transformation into TiO_x is located at the cathode region only for $SrTiO_3$:Nb, while for undoped $SrTiO_3$ almost the entire free surface transformed as can be seen from EDX analysis (Fig. S10b). Also the thickness of the nanoporous layer is much higher for undoped $SrTiO_3$ after comparable degradation time as revealed by SEM cross section analysis (Fig. S10c). These differences in transformation kinetics could be understood taking into account the higher ionic conductivity and the effect of localized conductivity[14] in undoped $SrTiO_3$. Since upon thermal reduction, oxygen is preferentially released from dislocations[15], the conductivity and non-equilibrium transport of oxygen of the reduced, but dislocation-rich surface layer will be higher than that of the bulk. In consequence, the stoichiometry polarization will be enhanced in the surface layer at the beginning of the transformation schematically illustrated in Fig. S10d. In the course of the decomposition, it can be assumed that a distorted interface layer is formed between the porous TiOx layer and the reduced bulk caused by the Sr sublimation. We thus expect that the ionic conductivity in this interface layer is increased compared to the bulk values due to the high concentration of vacancies. This could support the establishment of the low oxygen activity in the interface layer which will be shifting from the original surface of the crystal towards the bulk in the course of decomposition. This way, an out-of-plane decomposition can be realized via an in-plane polarization.

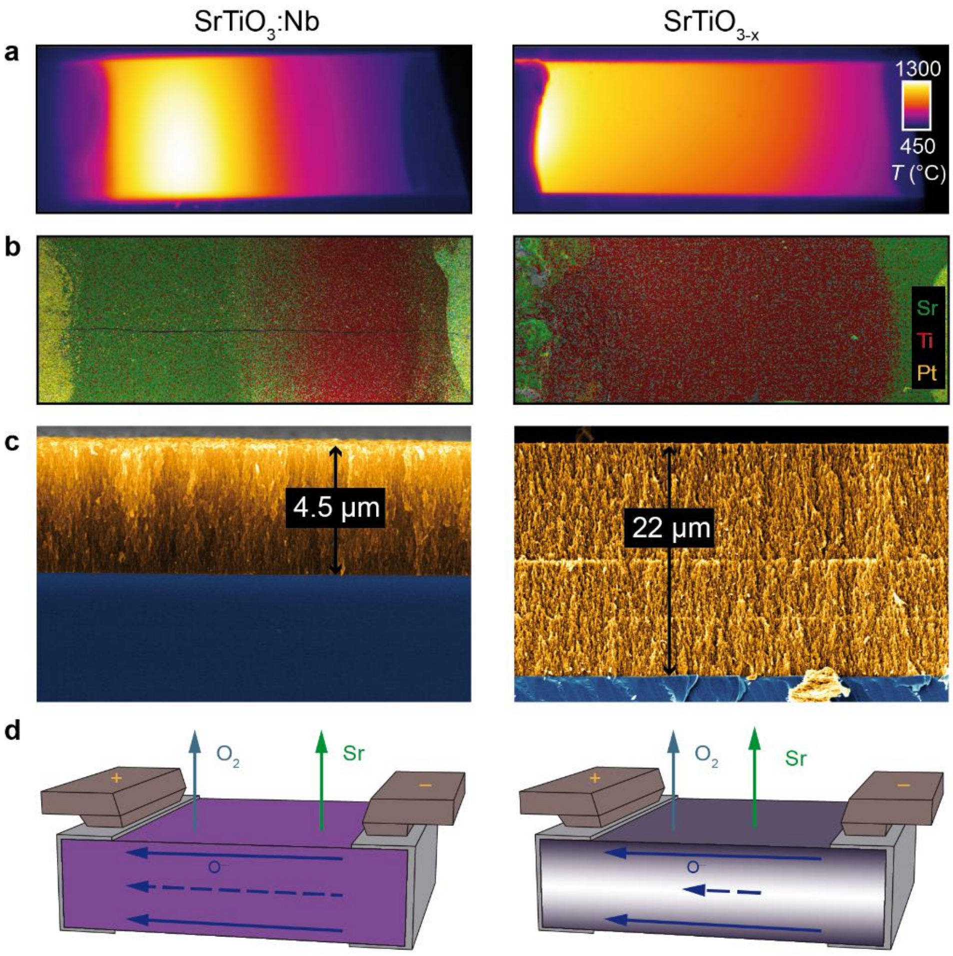

Figure S10: Comparison of electrochemical transformation in Nb-doped and undoped $SrTiO_3$. a) Infrared thermography during polarization using a constant current of 2.5 A. b) EDX Mapping and c) SEM cross section after 18 h degradation time. d) Illustration of the assumed oxygen flow during stoichiometry polarization.

IIIc. LOCALIZED CONDUCTIVITY IN $SrTiO_3$

In order to illustrate the effect of localized conductivity, LC-AFM experiments were performed. An undoped $SrTiO_3$ sample was reduced at 900 °C for 1 h and subsequently polarized by a DC voltage under UHV conditions for 5 min. This way, we aimed to investigate the first stage of electrodegradation, in which the macroscopic decomposition has not set in yet. After this treatment, the crystal was cleaved and the edge of the fracture was analysed by LC-AFM. The resulting topography and current maps are shown in Fig. S11. Since $SrTiO_3$ does not have a cleavage plane due to the high symmetry of the perovskite structure, the topography reveals a very rough surface with conchoidal morphology. The corresponding current maps show that the conductivity was highly inhomogeneous consisting of highly conducting areas with dimensions of a few tens of nanometres within a low-conducting surrounding. These findings support the idea that the electronic conductivity of $SrTiO_3$ has a localized nature presumably caused by the presence of dislocations[14], at least in the first stage of electrodegradation. This illustrates that the thermodynamic model presented in the manuscript assuming incongruent Sr sublimation due to the low oxygen activity established by electrochemical stoichiometry polarization can only serve as a first approach in understanding the underlying driving forces but we are aware that this cannot reproduce all the complex effects of nanoscale current channelling whose investigation remains a challenging task for future research and lays outside the scope of the present paper.

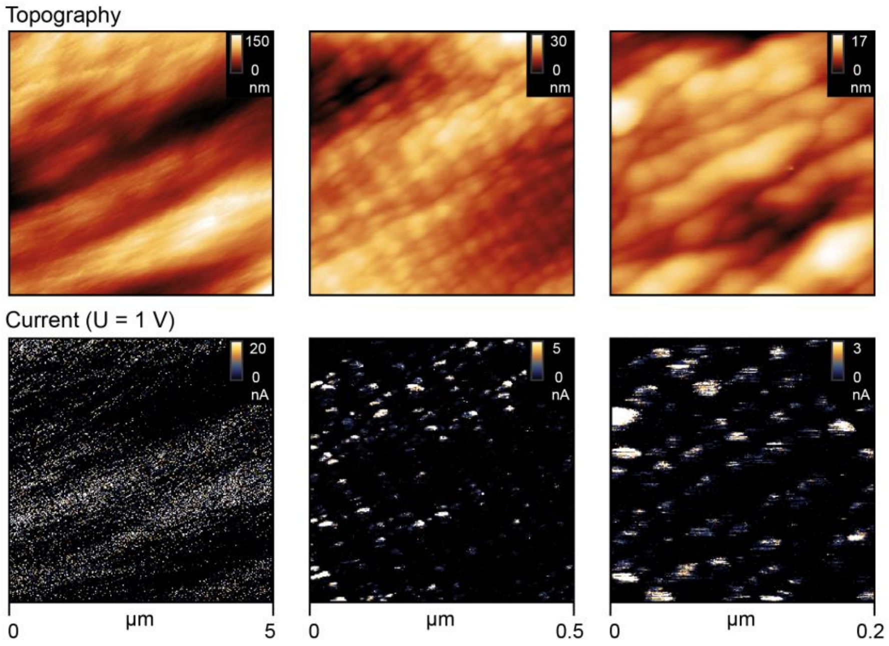

Figure S11. LC-AFM investigation of a short-time electrodegraded $SrTiO_3$ crystal after fracturing.

REFERENCES

1. Rodenbücher, C. *et al.* Stability and decomposition of perovskite-type titanates upon high-temperature reduction. *Phys. Status Solidi RRL* **11**, 1700222 (2017).
2. Vasquez, R. X-ray photoelectron spectroscopy study of Sr and Ba compounds. *Journal of Electron Spectroscopy and Related Phenomena* **56**, 217-240 (1991).
3. Szot, K., Speier, W., Herion, J. & Freiburg, C. Restructuring of the surface region in $SrTiO_3$. *Appl. Phys. A* **A64**, 55-9 (1997).
4. Baeumer, C. *et al.* Verification of redox-processes as switching and retention failure mechanisms in Nb:$SrTiO_3$/metal devices. *Nanoscale* **8**, 13967-13975 (2016).
5. Smyth, D. M. *The defect chemistry of metal oxides* (Oxford University Press, 2000).
6. Moos, R. & Härdtl, K. H. Defect chemistry of donor-doped and undoped strontium titanate ceramics between 1000°C and 1400°C. *J. Am. Ceram. Soc.* **80**, 2549-62 (1997).
7. Franco, M. A. A. & Regi, M. V. Anion deficiency in strontium titanate. *Nature* **270**, 706-707 (1977).
8. Leisegang, T. *et al.* Switching Ti Valence in $SrTiO_3$ by a dc Electric Field. *Phys. Rev. Lett.* **102**, 87601/1-4 (2009).
9. Andersson, S., Collén, B., Kuylenstierna, U. & Magnéli, A. Phase analysis studies on the titanium-oxygen system. *Acta Chemica Scandinavica* **11**, 1641-1652 (1957).
10. Perdew, J. P., Burke, K. & Ernzerhof, M. Generalized Gradient Approximation Made Simple. *Phys. Rev. Lett.* **77**, 3865-3868 (1996).
11. Slipukhina, I. & Lezaic, M. Electronic and magnetic properties of the Ti_5O_9 Magnéli phase. *Phys. Rev. B: Condens. Matter* **90**, 155133/1- (2014).
12. Guo, Y., Clark, S. J. & Robertson, J. Electronic and magnetic properties of Ti_2O_3, Cr_2O_3, and Fe_2O_3 calculated by the screened exchange hybrid density functional. *J. Phys. Condens. Mat.* **24**, 325504/1- (2012).
13. Neckel, A., Rastl, P., Eibler, R., Weinberger, P. & Schwarz, K. Results of self-consistent band-structure calculation for ScN, ScO, TiC, TiN, TiO, VC, VN and VO. *Journal of Physics C-Solid State Physics* **9**, 579-592 (1976).
14. Szot, K., Speier, W., Carius, R., Zastrow, U. & Beyer, W. Localized metallic conductivity and self-healing during thermal reduction of $SrTiO_3$. *Phys. Rev. Lett.* **88**, 075508/1-4 (2002).
15. Marrocchelli, D., Sun, L. & Yildiz, B. Dislocations in $SrTiO_3$: Easy To Reduce but Not so Fast for Oxygen Transport. *J. Am. Chem. Soc.* **137**, 4735-4748 (2015).
